# Supplementary material for: lncRNA CARINH regulates expression and function of innate immune transcription factor IRF1 in macrophages
Source: Life Sci Alliance. 2025 Jan 7;8(3):e202403021. doi: 10.26508/lsa.202403021 (PMC11707381; doi:10.26508/lsa.202403021)
Supplement: Supplementary file 1 [file LSA-2024-03021_TableS1.docx]

**Supplemental Material**

Table S1. LncRNAs differentially expressed in MPV, IAV and CoV2 vs healthy controls

| **MPV versus healthy controls** | | |
| --- | --- | --- |
| **geneID** | **Log2FoldChange** | **Padj** |
| MIR4435-2HG | 1.979826922 | 1.76E-17 |
| MIR600HG | -1.923022598 | 3.79E-16 |
| MIR3945HG | 3.332765952 | 7.63E-14 |
| CYTOR | 1.732755973 | 1.37E-13 |
| RARA-AS1 | 1.661681128 | 3.11E-12 |
| DBH-AS1 | -2.376664652 | 1.32E-10 |
| PCBP1-AS1 | 1.91133104 | 3.81E-10 |
| LINC01410 | 1.460751493 | 6.42E-10 |
| LINC01093 | 3.857852123 | 8.56E-10 |
| LINC00861 | -1.158554339 | 2.68E-09 |
| GSEC | 2.740870659 | 2.90E-09 |
| APOBEC3B-AS1 | 1.58168959 | 6.15E-09 |
| LINC01215 | -1.852497211 | 6.37E-09 |
| LINC00987 | -1.119676067 | 6.74E-09 |
| LINC00667 | -1.008901878 | 1.43E-08 |
| LINC01259 | -2.842109496 | 1.81E-08 |
| CCR5AS | 2.34019307 | 2.41E-08 |
| LINC01001 | 1.500199341 | 3.70E-08 |
| LINC01550 | -1.825828396 | 3.99E-08 |
| LINC00482 | 1.896487071 | 4.24E-08 |
| LINC01270 | 2.10982699 | 8.67E-08 |
| ADAMTSL4-AS1 | 1.9993858 | 1.29E-07 |
| CFAP58-DT | 2.446724684 | 1.48E-07 |
| LINC01184 | -1.008073453 | 1.51E-07 |
| DHRS4-AS1 | -0.949670221 | 2.33E-07 |
| HCG27 | 1.427938981 | 2.47E-07 |
| DLEU2 | 1.285071346 | 2.83E-07 |
| LINC02207 | 2.794293709 | 3.57E-07 |
| LINC01506 | 1.651787391 | 4.35E-07 |
| CARINH | 1.599751029 | 5.94E-07 |
| EPB41L4A-AS1 | -1.138403245 | 5.96E-07 |
| PKD1P6-NPIPP1 | -0.854957696 | 7.03E-07 |
| PSMB8-AS1 | 0.780653939 | 7.48E-07 |
| LINC02422 | 1.925591683 | 8.54E-07 |
| HCP5 | 0.828111437 | 1.08E-06 |
| RERE-AS1 | 1.840356698 | 1.20E-06 |
| BISPR | 1.685807651 | 1.20E-06 |
| CEROX1 | -1.395011833 | 1.55E-06 |
| SNHG29 | -0.935295733 | 1.91E-06 |
| SLC25A25-AS1 | -1.117416975 | 2.03E-06 |
| LINC02352 | 1.231877829 | 2.33E-06 |
| SNHG32 | -0.904453333 | 2.46E-06 |
| LINC02656 | 2.470621424 | 2.58E-06 |
| LINC01531 | 1.946402755 | 3.51E-06 |
| LINC01002 | 1.497215988 | 3.75E-06 |
| MDS2 | -1.692002027 | 4.72E-06 |
| FAM30A | -1.385077033 | 4.75E-06 |
| LINC00173 | 1.480450763 | 5.04E-06 |
| LINC01762 | 2.532168845 | 5.27E-06 |
| SATB1-AS1 | -2.141610553 | 6.47E-06 |
| PATL2 | 0.974219271 | 1.16E-05 |
| LINC00649 | -1.349577146 | 1.53E-05 |
| NRIR | 2.737219798 | 1.79E-05 |
| CACNA1C-AS1 | -1.763782022 | 2.10E-05 |
| LINC01127 | 1.953071723 | 2.24E-05 |
| KRT73-AS1 | -2.128390383 | 2.24E-05 |
| FAM13A-AS1 | 1.086649054 | 2.52E-05 |
| WASIR2 | -2.513726673 | 2.52E-05 |
| FAM157C | 1.31263741 | 2.75E-05 |
| LINC00937 | 1.35309706 | 2.86E-05 |
| NEAT1 | 1.393305424 | 3.04E-05 |
| LINC00211 | 1.667233349 | 3.07E-05 |
| ARHGAP27P1-BPTFP1-KPNA2P3 | 0.563657948 | 3.13E-05 |
| LINC00926 | -1.31246 | 3.25E-05 |
| LINC01347 | 1.037893739 | 3.49E-05 |
| HOTAIRM1 | 1.338911162 | 3.97E-05 |
| SIRPG-AS1 | -2.226477581 | 4.64E-05 |
| TSPOAP1-AS1 | -0.945797548 | 4.81E-05 |
| LINC01138 | 0.863282376 | 5.06E-05 |
| LINC01303 | 2.097370022 | 5.56E-05 |
| LINC01588 | -0.908838543 | 5.75E-05 |
| LINC02648 | -1.586657453 | 6.09E-05 |
| NFE4 | 1.686810234 | 6.16E-05 |
| HOXB-AS1 | -1.652438024 | 6.44E-05 |
| SNHG14 | -1.659436524 | 7.70E-05 |
| CARD8-AS1 | 0.82888845 | 8.29E-05 |
| LINC02649 | 2.024551874 | 8.31E-05 |
| LINC00528 | 0.643573 | 0.000101957 |
| C5orf67 | 2.24060697 | 0.000111935 |
| MAP3K14 | -0.586108153 | 0.000144626 |
| RNF213-AS1 | 1.764658186 | 0.00015736 |
| BASP1-AS1 | 2.246551147 | 0.000168722 |
| MCF2L-AS1 | -2.248905842 | 0.000195087 |
| LINC02471 | 2.56521583 | 0.000198854 |
| SNHG1 | -0.685766451 | 0.000219272 |
| MICB-DT | 0.989857975 | 0.000243608 |
| PIK3CD-AS1 | 0.977459951 | 0.000263048 |
| PSMD6-AS2 | 0.950693155 | 0.000289859 |
| PDXDC2P-NPIPB14P | -0.702291181 | 0.000303562 |
| LINC00671 | 1.937831664 | 0.000317043 |
| LINC02528 | 2.410002425 | 0.000364007 |
| HLA-DQB1-AS1 | -1.231099634 | 0.000481582 |
| FAM153CP | -1.490605134 | 0.000494334 |
| LINC01128 | -0.70943788 | 0.000497771 |
| A1BG-AS1 | -1.088947309 | 0.000529457 |
| ITPK1-AS1 | 1.336360186 | 0.000545959 |
| LINC01678 | -0.933906637 | 0.000581735 |
| LINC01278 | -0.669345625 | 0.000608975 |
| SNHG7 | -0.578197463 | 0.000627474 |
| PCED1B-AS1 | -0.713259434 | 0.000627474 |
| HEIH | 0.719486449 | 0.000712513 |
| HCG18 | -0.648599395 | 0.0007599 |
| LINC01094 | 1.694313387 | 0.000789475 |
| ZFAS1 | 0.786276235 | 0.00083677 |
| NCF4-AS1 | 1.396001659 | 0.000897441 |
| NDUFA6-DT | -0.846721762 | 0.000912338 |
| LINC01353 | 1.906200222 | 0.001240295 |
| KMT2E-AS1 | 0.557215404 | 0.00125152 |
| NUTM2A-AS1 | 0.876870043 | 0.001337502 |
| DISC1-IT1 | 2.200508076 | 0.0013722 |
| FAM27E3 | 1.792314237 | 0.001428459 |
| GAS5 | -0.729096454 | 0.001435125 |
| LINC02217 | 2.129493892 | 0.00155051 |
| GABPB1-AS1 | -0.788626753 | 0.001563357 |
| CYP1B1-AS1 | 1.717010172 | 0.0016012 |
| MIR210HG | -1.889843898 | 0.001651012 |
| LINC01891 | -1.858207545 | 0.001685088 |
| LUCAT1 | 0.784477864 | 0.001723348 |
| CFLAR-AS1 | 1.437751716 | 0.001760725 |
| WASIR1 | -2.153151688 | 0.001770861 |
| LINC02615 | -1.95341473 | 0.001843043 |
| LINC02067 | -0.771822581 | 0.001910015 |
| ILF3-DT | -0.639051118 | 0.002176041 |
| SENCR | 0.717704359 | 0.002562527 |
| LINC00487 | 2.016103854 | 0.002641455 |
| PRKCQ-AS1 | -0.929356201 | 0.002673104 |
| RORA-AS1 | -1.367234418 | 0.002746568 |
| LINC01163 | -1.331600456 | 0.002806543 |
| FAM41C | 1.954987184 | 0.002968923 |
| TRG-AS1 | -0.714945425 | 0.002993477 |
| CTBP1-DT | -0.754834765 | 0.003136113 |
| LINC01485 | 1.946958003 | 0.00322589 |
| SLC12A5-AS1 | 2.048553618 | 0.003311111 |
| SNHG17 | -0.829610164 | 0.003463523 |
| A2M-AS1 | -1.606750213 | 0.00355886 |
| LINC00402 | -1.561547414 | 0.003570253 |
| LOH12CR2 | 1.808706348 | 0.003570546 |
| DLGAP1-AS2 | 1.396130648 | 0.003846001 |
| CHRM3-AS2 | -1.673800796 | 0.003888456 |
| LINC00342 | -1.0093776 | 0.003928569 |
| LINC02361 | -0.824159984 | 0.004001778 |
| LINC-PINT | 0.780902737 | 0.004030603 |
| LINC01988 | 1.617009715 | 0.004106113 |
| KCNJ2-AS1 | 1.582395862 | 0.004136184 |
| LINC00623 | 0.674974338 | 0.004192142 |
| LINC00963 | 0.677876404 | 0.00444052 |
| LINC01281 | -2.021232271 | 0.004599752 |
| PKN2-AS1 | 1.938643839 | 0.004658088 |
| IL10RB-DT | 0.848572727 | 0.004767525 |
| LINC02878 | -1.545247311 | 0.004785977 |
| LINC01521 | -1.070162591 | 0.004799197 |
| NSMCE1-DT | 1.629238852 | 0.004840446 |
| C1orf220 | -1.508013806 | 0.004857155 |
| LINC02363 | 1.558369043 | 0.004872312 |
| LINC02205 | 2.002444756 | 0.004937029 |
| DLGAP1-AS1 | 1.007773234 | 0.005294294 |
| LINC00996 | -1.284060366 | 0.005367584 |
| LINC01238 | -1.351100256 | 0.005463959 |
| OBSCN-AS1 | -1.672482091 | 0.005619917 |
| PTCSC1 | 1.473803746 | 0.006077808 |
| MROCKI | 1.952525056 | 0.006144449 |
| GSN-AS1 | 1.425413561 | 0.006156763 |
| LINC01260 | -1.100740775 | 0.006167626 |
| OR2A1-AS1 | -1.497655295 | 0.006271888 |
| LINC01089 | -0.611240014 | 0.006464259 |
| LINC01503 | 1.072062056 | 0.006503206 |
| PSORS1C3 | 1.775793999 | 0.00655963 |
| LINC00638 | -1.69657294 | 0.006577665 |
| LINC02273 | -1.19754564 | 0.006579818 |
| DANCR | -0.730137804 | 0.006827527 |
| TAPT1-AS1 | -0.652903933 | 0.007199668 |
| CKMT2-AS1 | -1.234239539 | 0.007297533 |
| VIPR1-AS1 | -1.605644842 | 0.007574159 |
| KLF3-AS1 | -0.812259625 | 0.007607215 |
| EBLN3P | -0.766424016 | 0.007868837 |
| LINC00174 | 0.685671257 | 0.008364735 |
| MZF1-AS1 | -0.837550157 | 0.008616786 |
| PANDAR | 1.711155861 | 0.008952784 |
| LINC01176 | 1.119565866 | 0.009326464 |
| ASAP1-IT2 | 1.377642335 | 0.009343434 |
| LINC00570 | 1.448103927 | 0.009453324 |
| LINC02068 | 1.896877028 | 0.009727724 |
| ATP6V0E2-AS1 | -1.187423682 | 0.010004086 |
| LINC02325 | -1.389504892 | 0.010123563 |
| ZNF32-AS2 | -1.72472846 | 0.010317356 |
| ELFN2 | -1.509561094 | 0.010861513 |
| LRRC8C-DT | -1.193026487 | 0.010955779 |
| LINC00205 | -1.049260656 | 0.011010927 |
| ITGB2-AS1 | 0.59159125 | 0.01113204 |
| LINC00612 | -1.717772948 | 0.011218657 |
| LINC01013 | -1.744896341 | 0.011233771 |
| PRR29-AS1 | -1.68932227 | 0.01141506 |
| SCARNA9 | 0.794210317 | 0.011531314 |
| LEF1-AS1 | -1.624782186 | 0.011600906 |
| PITRM1-AS1 | -1.081331319 | 0.011651875 |
| ENTPD1-AS1 | 1.241142739 | 0.012262867 |
| LINC02289 | 1.047535549 | 0.01234926 |
| SNHG8 | -0.852198156 | 0.012435248 |
| LINC00663 | -0.803773571 | 0.012574798 |
| PVT1 | -0.559341503 | 0.012956098 |
| LINC02397 | -1.264368661 | 0.01298865 |
| LINC01934 | -1.269056536 | 0.013259165 |
| CCDC183-AS1 | -1.171639968 | 0.014282293 |
| LINC00877 | 1.018771895 | 0.015119725 |
| HLA-F-AS1 | 1.081722486 | 0.015651169 |
| LINC00899 | 0.649470882 | 0.01567921 |
| EIF1B-AS1 | 1.266404769 | 0.01594389 |
| MHENCR | -0.60200656 | 0.016000485 |
| MATN1-AS1 | -1.189987211 | 0.016047804 |
| LINC01300 | 1.773171243 | 0.016242975 |
| FAM66C | -1.495497864 | 0.016280746 |
| RAD51-AS1 | -1.015454243 | 0.016656527 |
| DOCK8-AS1 | 1.087694107 | 0.016718582 |
| BFSP2-AS1 | -1.406924388 | 0.016769267 |
| SNHG3 | -0.354944066 | 0.016850579 |
| PRNCR1 | -0.633568113 | 0.017339568 |
| GUSBP11 | 0.443414494 | 0.01748572 |
| PLBD1-AS1 | 1.433533662 | 0.017548347 |
| HSD11B1-AS1 | 1.565661045 | 0.0184021 |
| AOAH-IT1 | 1.360339679 | 0.018469725 |
| CLCA4-AS1 | 1.488095621 | 0.018505478 |
| SNHG19 | -1.063241971 | 0.018747697 |
| TRPM2-AS | 1.419615339 | 0.018825656 |
| PSMB1 | 0.474685401 | 0.019286344 |
| DNAJC27-AS1 | 1.328798332 | 0.019518771 |
| HMGA2-AS1 | 1.620133341 | 0.020503232 |
| PCCA-DT | -1.535643801 | 0.021108087 |
| LINC01271 | 1.451722614 | 0.021132878 |
| VPS9D1-AS1 | 0.946205228 | 0.02150485 |
| MIR762HG | -0.955860832 | 0.021591325 |
| LINC00664 | 1.591786569 | 0.021954496 |
| LINC01191 | 1.297299191 | 0.022371283 |
| CACNA1C-AS2 | -1.255152626 | 0.022654851 |
| MMP25-AS1 | 0.568085971 | 0.022670077 |
| LINC01006 | -0.947881301 | 0.022777144 |
| LINC00654 | 1.158591866 | 0.023702864 |
| LIPC-AS1 | 1.582164899 | 0.023814579 |
| JPX | 1.020274713 | 0.023989335 |
| HIF1A-AS3 | 1.48007912 | 0.024240034 |
| MKNK1-AS1 | 1.334797687 | 0.024556693 |
| NINJ2-AS1 | 0.580470567 | 0.024832364 |
| LINC00526 | -1.24392889 | 0.025338346 |
| MAP3K5-AS1 | 1.218909205 | 0.025470332 |
| LPP-AS2 | 1.351021489 | 0.026506317 |
| LINC01355 | -0.920733881 | 0.027227039 |
| LINC02611 | -0.822357441 | 0.027299765 |
| LINC00685 | -0.471775569 | 0.027323594 |
| LINC00886 | -1.316617526 | 0.027601974 |
| LINC02446 | -1.073650698 | 0.029242381 |
| ARF4-AS1 | 1.162073046 | 0.029483023 |
| LINC00862 | 1.34687554 | 0.031460678 |
| TCL6 | -1.327489881 | 0.031664092 |
| ATP11A-AS1 | 1.19176677 | 0.032092092 |
| TLR8-AS1 | 1.556445182 | 0.032435364 |
| LINC00920 | -1.239898587 | 0.034291676 |
| STXBP5-AS1 | 1.17747385 | 0.03464651 |
| SCAT8 | 1.108639928 | 0.035290671 |
| FAM225A | 1.322084525 | 0.035627752 |
| LINC00930 | 1.53383991 | 0.035699245 |
| LINC02033 | 1.058167022 | 0.036404875 |
| SNHG6 | -0.481938919 | 0.037093644 |
| DLEU1 | 0.736273688 | 0.037257059 |
| SLC25A5-AS1 | -1.053209424 | 0.037476414 |
| CHROMR | 1.104130395 | 0.038901182 |
| LINC01237 | -0.985211432 | 0.039333563 |
| LINC02245 | -1.462955392 | 0.039416629 |
| SPATA13 | 0.632063699 | 0.039622937 |
| RNF157-AS1 | -1.227438745 | 0.040362955 |
| LY6E-DT | 1.432170238 | 0.040860137 |
| PPP3CB-AS1 | -0.545706542 | 0.041516212 |
| LINC00954 | -0.707104439 | 0.042235763 |
| RNASEH1-AS1 | -0.872938559 | 0.04244887 |
| TPT1-AS1 | 0.455079639 | 0.042897144 |
| LINC01137 | 0.834554951 | 0.043617608 |
| LINC02574 | 1.494635811 | 0.043974732 |
| CASC8 | -1.504201687 | 0.045819647 |
| LINC01136 | 1.16228015 | 0.047151027 |
| DICER1-AS1 | 0.821290537 | 0.048960204 |
| LINC01635 | -1.37566635 | 0.049270288 |
| LINC00865 | -1.085705142 | 0.049523669 |
| NRAV | -0.755056448 | 0.049783222 |

| **IAV versus healthy controls** | | |
| --- | --- | --- |
| **geneID** | **Log2FoldChange** | **Padj** |
| CCR5AS | 2.916821858 | 1.53E-23 |
| BISPR | 1.847858374 | 1.80E-22 |
| NRIR | 3.353648994 | 4.87E-22 |
| LINC02528 | 3.963867865 | 1.62E-20 |
| CFAP58-DT | 2.495532124 | 4.05E-19 |
| APOBEC3B-AS1 | 1.830280811 | 4.98E-19 |
| CARINH | 1.90350956 | 2.99E-18 |
| RNF213-AS1 | 2.272604643 | 1.39E-16 |
| LINC01531 | 2.568013581 | 1.69E-16 |
| MIR3945HG | 2.760622886 | 2.06E-16 |
| LINC01410 | 1.451607418 | 5.84E-16 |
| LINC02471 | 3.421896665 | 9.24E-16 |
| MIR4435-2HG | 1.358955172 | 4.67E-15 |
| FAM225B | 2.523995164 | 6.36E-15 |
| FAM225A | 2.550067923 | 7.31E-14 |
| LINC00487 | 2.966824319 | 7.39E-14 |
| LINC02422 | 1.876918827 | 8.08E-14 |
| PANDAR | 2.689525144 | 8.25E-14 |
| CABIN1 | -0.884875828 | 1.57E-13 |
| LINC01504 | 1.537552652 | 2.43E-13 |
| PCBP1-AS1 | 1.576101808 | 4.92E-13 |
| LINC01093 | 3.448418054 | 7.99E-13 |
| PPM1K-DT | 3.37070662 | 8.09E-13 |
| LINC00482 | 1.355221567 | 9.71E-13 |
| AATBC | 1.365048958 | 1.04E-12 |
| CYTOR | 1.350291987 | 2.16E-12 |
| TNK2-AS1 | 1.565310006 | 7.97E-12 |
| ADAMTSL4-AS1 | 1.727534771 | 8.00E-12 |
| LINC01887 | 2.677945519 | 2.42E-11 |
| RERE-AS1 | 1.621103517 | 2.61E-11 |
| USP30-AS1 | 1.305151128 | 3.77E-11 |
| SNHG29 | -0.836922225 | 4.62E-11 |
| MHENCR | -0.999562353 | 1.40E-10 |
| PKN2-AS1 | 2.496043126 | 1.52E-10 |
| LINC01094 | 2.138107904 | 1.52E-10 |
| CHROMR | 1.88395396 | 1.58E-10 |
| JPX | 1.329334994 | 2.57E-10 |
| LINC01089 | -0.86823591 | 3.39E-10 |
| GSN-AS1 | 2.028026335 | 4.79E-10 |
| DISC1-IT1 | 2.782179389 | 1.04E-09 |
| LINC02068 | 3.174170063 | 1.13E-09 |
| LINC01679 | 1.68734968 | 1.28E-09 |
| LINC00667 | -0.642583553 | 5.33E-09 |
| TSPOAP1-AS1 | -0.707597426 | 6.60E-09 |
| LINC01550 | -1.282265898 | 7.20E-09 |
| HCP5 | 0.606018856 | 7.84E-09 |
| CARD8-AS1 | 0.841580518 | 1.04E-08 |
| SERPINB9P1 | 1.787968415 | 1.52E-08 |
| LINC00921 | -0.699050232 | 1.67E-08 |
| ZEB2-AS1 | 1.474263257 | 2.25E-08 |
| RARA-AS1 | 0.989182684 | 3.11E-08 |
| MIR600HG | -1.289669127 | 3.25E-08 |
| AOAH-IT1 | 1.679911605 | 3.28E-08 |
| MDS2 | -1.358469082 | 3.45E-08 |
| MICB-DT | 0.99027518 | 4.22E-08 |
| FIRRE | 1.650987788 | 5.95E-08 |
| LINC01506 | 1.526176395 | 6.14E-08 |
| LINC00957 | -0.783764314 | 6.26E-08 |
| MGC16275 | 1.284484978 | 7.07E-08 |
| LINC01138 | 0.762521286 | 1.06E-07 |
| DLEU2 | 1.101795434 | 1.20E-07 |
| SLC25A25-AS1 | -0.874073626 | 1.20E-07 |
| LINC01184 | -0.722103972 | 1.53E-07 |
| LINC01521 | -1.265731931 | 1.90E-07 |
| GSEC | 1.599299602 | 2.19E-07 |
| LINC00900 | 2.070678269 | 2.74E-07 |
| SCAMP1-AS1 | 1.302516225 | 3.25E-07 |
| XXYLT1-AS2 | 2.068475864 | 3.97E-07 |
| LINC02574 | 2.442309103 | 4.24E-07 |
| DHRS4-AS1 | -0.709810035 | 5.39E-07 |
| PTGER4P2-CDK2AP2P2 | 1.614935251 | 5.67E-07 |
| LINC00968 | 1.759509254 | 6.43E-07 |
| HOXB-AS1 | -1.36521437 | 6.47E-07 |
| LINC01215 | -1.264064408 | 7.46E-07 |
| CCDC140 | 2.785171059 | 8.03E-07 |
| DOCK4-AS1 | 2.536221643 | 8.47E-07 |
| PRKCZ-AS1 | -0.984196665 | 9.67E-07 |
| C5orf67 | 1.862487684 | 1.21E-06 |
| LINC02878 | -1.931821422 | 1.27E-06 |
| LINC00861 | -0.782499084 | 1.80E-06 |
| PSMB8-AS1 | 0.636477771 | 1.92E-06 |
| SNHG8 | -0.863482093 | 2.04E-06 |
| LGALS8-AS1 | 1.873726197 | 2.44E-06 |
| DOCK8-AS1 | 1.314228401 | 2.94E-06 |
| KLF3-AS1 | -1.109512632 | 2.98E-06 |
| LINC02361 | -0.812156564 | 3.21E-06 |
| MIR9-3HG | -1.188104209 | 3.51E-06 |
| LINC02067 | -0.657343337 | 5.05E-06 |
| LINC02035 | -0.793510149 | 6.03E-06 |
| SENCR | 0.854984142 | 7.87E-06 |
| PIK3CD-AS1 | 0.743266919 | 8.62E-06 |
| GAS5 | -0.68531438 | 1.11E-05 |
| MIR34AHG | 1.781003099 | 1.16E-05 |
| HCG27 | 0.743908031 | 1.25E-05 |
| ELFN2 | -1.565238262 | 1.29E-05 |
| ATP6V0E2-AS1 | -1.218579057 | 1.49E-05 |
| LY6E-DT | 1.733940071 | 1.64E-05 |
| NUCB1-AS1 | 1.578333719 | 1.65E-05 |
| LINC01762 | 1.546928415 | 1.68E-05 |
| MIRLET7BHG | -0.631542819 | 1.76E-05 |
| OLMALINC | -1.208253475 | 1.77E-05 |
| LINC01259 | -1.619842542 | 1.98E-05 |
| SNHG32 | -0.601993457 | 2.35E-05 |
| DBH-AS1 | -1.365558341 | 2.66E-05 |
| SIRPG-AS1 | -1.61882616 | 2.71E-05 |
| TRIM52-AS1 | -0.800203732 | 3.25E-05 |
| NEAT1 | 0.874268851 | 3.61E-05 |
| LINC01011 | 0.995518703 | 4.50E-05 |
| LINC02033 | 1.269644292 | 4.74E-05 |
| LINC00877 | 0.937708711 | 4.79E-05 |
| ST3GAL5-AS1 | 1.495845666 | 4.86E-05 |
| SPATA13 | 0.835598087 | 5.42E-05 |
| LINC01359 | 1.381489512 | 5.43E-05 |
| LINC01303 | 1.283341399 | 5.49E-05 |
| LINC01278 | -0.420538631 | 6.29E-05 |
| RAD51-AS1 | -0.893441544 | 6.49E-05 |
| HOTAIRM1 | 0.719345842 | 6.52E-05 |
| LINC01353 | 1.33638993 | 7.61E-05 |
| HLA-F-AS1 | 0.876885355 | 8.15E-05 |
| EPB41L4A-AS1 | -0.643458737 | 8.31E-05 |
| OBSCN-AS1 | -1.48894116 | 8.31E-05 |
| CEROX1 | -0.850519198 | 8.56E-05 |
| ILF3-DT | -0.520163467 | 8.64E-05 |
| HOMER3-AS1 | 1.21108284 | 8.77E-05 |
| LINC02363 | 1.340419204 | 8.96E-05 |
| PTOV1-AS2 | -0.403707453 | 9.46E-05 |
| LBX2-AS1 | 0.940678536 | 0.000117243 |
| LCMT1-AS1 | 1.331987439 | 0.00011814 |
| LINC01006 | -1.016278256 | 0.000126554 |
| PSORS1C3 | 1.638692478 | 0.000133077 |
| TTC28-AS1 | -0.637758893 | 0.000137007 |
| WASIR2 | -1.731603908 | 0.000139676 |
| SNHG16 | -0.369895312 | 0.000141264 |
| CACNA1C-AS1 | -0.999740106 | 0.000144449 |
| LINC00847 | 0.750627432 | 0.000151473 |
| CFLAR-AS1 | 1.135763994 | 0.00015367 |
| KCNJ2-AS1 | 1.236039689 | 0.000174114 |
| PCED1B-AS1 | -0.560211536 | 0.000189983 |
| TLR8-AS1 | 1.698048733 | 0.00019999 |
| VIPR1-AS1 | -1.448825634 | 0.000230419 |
| LINC02212 | 1.323838474 | 0.000233683 |
| UCKL1-AS1 | 0.897086125 | 0.000236105 |
| OVOL1-AS1 | 2.012231194 | 0.000247335 |
| MAP3K14 | -0.323479472 | 0.000266089 |
| KRT73-AS1 | -1.544522669 | 0.00028343 |
| BCRP3 | -1.511164706 | 0.000288621 |
| FOXN3-AS1 | 0.912890368 | 0.000294273 |
| CAHM | 0.895265363 | 0.000299795 |
| MCF2L-AS1 | -1.551828956 | 0.000301448 |
| SLC9A3-AS1 | -0.997378704 | 0.000301684 |
| PINK1-AS | -0.698372754 | 0.000318051 |
| LINC02656 | 1.283471214 | 0.000325678 |
| GABPB1-AS1 | -0.743343338 | 0.000333735 |
| NRAV | -0.972148109 | 0.00033485 |
| HMGA2-AS1 | 1.630190241 | 0.000340443 |
| LINC00987 | -0.483761702 | 0.000351791 |
| SNHG6 | -0.455678222 | 0.000364451 |
| LINC00494 | -1.221506616 | 0.000382629 |
| PATL2 | 0.570811568 | 0.000431321 |
| PSMD6-AS2 | 0.751305989 | 0.000448404 |
| LMNB1-DT | 2.052816482 | 0.000451744 |
| DANCR | -0.565770907 | 0.000463089 |
| WASIR1 | -1.992405769 | 0.000481121 |
| PDXDC2P-NPIPB14P | -0.544184775 | 0.000521237 |
| ST20-AS1 | -0.663264874 | 0.000522347 |
| LINC00205 | -0.803445509 | 0.000552304 |
| ERICH6-AS1 | -1.069023124 | 0.000556325 |
| IL10RB-DT | 0.57786135 | 0.000557732 |
| NDUFA6-DT | -0.64524326 | 0.00057434 |
| PRKCQ-AS1 | -0.713095857 | 0.000611794 |
| SNHG9 | -0.731704521 | 0.000636141 |
| SATB1-AS1 | -1.194421571 | 0.000679749 |
| TPRG1-AS1 | 1.319774847 | 0.000744801 |
| RNASEH2B-AS1 | 1.522171249 | 0.000746006 |
| LINC02724 | 0.909091651 | 0.000752933 |
| HEIH | 0.651972881 | 0.000758034 |
| PRKAG2-AS1 | -0.942964002 | 0.000773945 |
| BAIAP2-DT | -0.524509595 | 0.000812684 |
| LINC00663 | -0.70892509 | 0.000877896 |
| DOCK9-DT | -1.029368062 | 0.0008847 |
| LINC01238 | -0.993542796 | 0.000886725 |
| LINC01232 | 0.77871033 | 0.000888532 |
| LINC01163 | -1.427839646 | 0.000927249 |
| NAPA-AS1 | 0.756793098 | 0.000938651 |
| KIFC1 | 1.107042943 | 0.000946404 |
| TTN-AS1 | 1.016506039 | 0.000950278 |
| LINC02273 | -0.862668826 | 0.000974679 |
| LINC01311 | -0.739779303 | 0.00098911 |
| LINC01819 | -1.594568661 | 0.001005548 |
| LINC02648 | -1.045502447 | 0.001068015 |
| LINC02384 | 0.997889689 | 0.001083361 |
| LINC02611 | -0.635016477 | 0.001107793 |
| CTBP1-DT | -0.499760184 | 0.001141463 |
| STK24-AS1 | 1.131306437 | 0.001176737 |
| FAM157C | 0.765385918 | 0.001199679 |
| CCDC183-AS1 | -0.768553449 | 0.001215545 |
| SNHG19 | -0.773345192 | 0.001235787 |
| LINC00189 | 1.294758365 | 0.001244221 |
| MAP3K14-AS1 | -0.685873798 | 0.001256208 |
| LINC00926 | -0.857748096 | 0.001267842 |
| LINC01671 | 1.490672076 | 0.001310713 |
| LINC00612 | -1.450782676 | 0.001324276 |
| TAPT1-AS1 | -0.507299408 | 0.001334858 |
| PSMB1 | 0.332159294 | 0.001374976 |
| LINC00342 | -0.784210106 | 0.001466404 |
| FAM153CP | -1.00582446 | 0.001519007 |
| SRGAP2-AS1 | 1.5092798 | 0.001542511 |
| PYCARD-AS1 | 0.71192033 | 0.001543982 |
| SNHG5 | -0.686570136 | 0.001554046 |
| GHRLOS | -0.535838798 | 0.001669862 |
| ARHGAP27P1-BPTFP1-KPNA2P3 | 0.365079415 | 0.0017064 |
| SNHG17 | -0.505815328 | 0.001844514 |
| CLEC12A-AS1 | 1.259655398 | 0.001909307 |
| EBLN3P | -0.560334776 | 0.001934428 |
| THAP9-AS1 | -0.381447058 | 0.001998252 |
| LINC00623 | 0.514822508 | 0.002055597 |
| MAP3K5-AS1 | 1.11962894 | 0.00211011 |
| DACT3-AS1 | 1.096759798 | 0.002170763 |
| LINC02085 | 1.298292147 | 0.002190714 |
| LINC01281 | -1.739991368 | 0.002214077 |
| LINC02649 | 0.959640427 | 0.002236827 |
| LEF1-AS1 | -1.377136823 | 0.002285122 |
| LINC01678 | -0.594744015 | 0.002348003 |
| LOXL1-AS1 | -0.839978444 | 0.002474186 |
| LINC02009 | 1.359707994 | 0.002511049 |
| ZNF213-AS1 | -0.428265056 | 0.002514824 |
| LINC02432 | 1.144255551 | 0.002530237 |
| LINC02158 | -0.938114818 | 0.002538135 |
| A1BG-AS1 | -0.58176269 | 0.002567319 |
| LINC02217 | 1.48439559 | 0.002788751 |
| LINC00476 | -0.524271493 | 0.002809442 |
| SH3BP5-AS1 | -0.502004638 | 0.002882662 |
| LINC00570 | 1.098396391 | 0.002946775 |
| LINC02352 | 0.622985945 | 0.002951835 |
| PSMG3-AS1 | -0.636325749 | 0.003039567 |
| PHF1 | -0.265607881 | 0.003062964 |
| LINC01176 | 0.70714381 | 0.003111762 |
| MIR210HG | -1.228363165 | 0.003164523 |
| OR2A1-AS1 | -1.081845395 | 0.003223087 |
| PLA2G4C-AS1 | 1.619006046 | 0.003243426 |
| LINC01485 | 1.356173954 | 0.003266264 |
| LINC02446 | -1.043426585 | 0.003281301 |
| MAN1B1-DT | -0.929505779 | 0.003386048 |
| HIF1A-AS3 | 1.253018197 | 0.003493851 |
| PKD1P6-NPIPP1 | -0.372504128 | 0.003493851 |
| MATN1-AS1 | -0.751463126 | 0.003522138 |
| PPP1R12A-AS1 | 1.27717135 | 0.00352687 |
| KRTAP5-AS1 | -1.482479138 | 0.003984112 |
| LINC01270 | 0.867422886 | 0.004193755 |
| LINC00243 | -0.693776864 | 0.004229925 |
| CTBP1-AS | -0.409036116 | 0.004316689 |
| LINC01588 | -0.507823027 | 0.004338123 |
| LINC00853 | 0.957442572 | 0.004424146 |
| NOP14-AS1 | -0.342927266 | 0.004580969 |
| LINC01300 | 1.628018816 | 0.004591103 |
| LINC01146 | 1.136695589 | 0.004829348 |
| ITPK1-AS1 | 0.758130994 | 0.00489409 |
| LINC01963 | -0.608013145 | 0.005122856 |
| RCAN3AS | -1.097122026 | 0.005137963 |
| LINC01128 | -0.433341677 | 0.005155045 |
| LINC01732 | 1.502918989 | 0.005192481 |
| MZF1-AS1 | -0.569768281 | 0.005240877 |
| LINC02421 | -1.646663304 | 0.005373606 |
| ABALON | 1.009952649 | 0.005922286 |
| IL6R-AS1 | -1.046041421 | 0.005989051 |
| STPG3-AS1 | -1.080155831 | 0.005997983 |
| LINC01619 | -1.062718205 | 0.006254491 |
| ZNF8-ERVK3-1 | -1.234817132 | 0.006319452 |
| SNHG1 | -0.34285497 | 0.006376482 |
| BASP1-AS1 | 1.293572235 | 0.006455418 |
| A2M-AS1 | -0.965164894 | 0.006476883 |
| SLC25A5-AS1 | -0.768798142 | 0.006568185 |
| LINC00526 | -0.87786099 | 0.006694423 |
| LINC00528 | 0.307019623 | 0.006763507 |
| ZNF667-AS1 | -0.909562576 | 0.006837462 |
| PRSS30P | -0.878502515 | 0.007345983 |
| SLC12A9-AS1 | -1.386279773 | 0.007413974 |
| SH3RF3-AS1 | -1.469253801 | 0.007744245 |
| KIF1C-AS1 | 0.808700116 | 0.007831067 |
| LINC00886 | -0.907506515 | 0.007862516 |
| SCARNA9 | 0.635178766 | 0.007896596 |
| SRRM2-AS1 | -1.153357131 | 0.007981723 |
| FLJ37453 | -0.304656507 | 0.008075102 |
| LINC00954 | -0.478052296 | 0.008075102 |
| TRPM2-AS | 1.013596457 | 0.00814427 |
| SNHG25 | -1.066556264 | 0.00835981 |
| TDRKH-AS1 | -0.949581385 | 0.008399472 |
| PRKAR1B-AS1 | 0.548458104 | 0.009135159 |
| ARMCX5-GPRASP2 | -1.066394712 | 0.00915448 |
| NUTM2A-AS1 | 0.552394802 | 0.009159953 |
| SEMA3B-AS1 | 1.360562793 | 0.009443869 |
| LINC01637 | 0.793247556 | 0.009535115 |
| SNHG14 | -0.756180937 | 0.009791865 |
| ATP11A-AS1 | 0.888221485 | 0.009899224 |
| EMC1-AS1 | -0.731064419 | 0.010477697 |
| LINC00294 | -0.325533848 | 0.01070018 |
| TTC39C-AS1 | 0.687034911 | 0.010895588 |
| LUNAR1 | -1.342596953 | 0.01092464 |
| FRY-AS1 | -0.88554563 | 0.010985389 |
| LINC02012 | -1.165265038 | 0.011006935 |
| LINC00852 | -0.651602142 | 0.011628174 |
| LINC01882 | -0.875570978 | 0.011645142 |
| LINC02362 | 0.946749056 | 0.01175991 |
| DNAJC27-AS1 | 0.897578019 | 0.011784338 |
| LINC01857 | -0.808772282 | 0.011979799 |
| CCDC18-AS1 | -0.510343365 | 0.012308321 |
| OGFR-AS1 | 0.758958542 | 0.012334872 |
| ARRDC1-AS1 | -0.260832307 | 0.012384688 |
| LINC00685 | -0.370232296 | 0.01242442 |
| ID2-AS1 | 0.772422287 | 0.012637214 |
| LINC01750 | 1.259099857 | 0.012812033 |
| LINC00649 | -0.548823093 | 0.013291446 |
| HLA-DQB1-AS1 | -0.634351273 | 0.013325327 |
| RFPL3S | 0.864787652 | 0.013464286 |
| SPAG16-DT | -1.551787446 | 0.013730963 |
| VPS9D1-AS1 | 0.582003031 | 0.014117549 |
| LINC00989 | 0.843091186 | 0.014286561 |
| LINC00115 | -0.526128177 | 0.014660953 |
| L3MBTL2-AS1 | 0.752271903 | 0.014862452 |
| LINC01237 | -0.697635353 | 0.014903381 |
| LINC01002 | 0.573279361 | 0.015060117 |
| PARD6G-AS1 | 1.222009973 | 0.015320471 |
| LINC02520 | -1.059904356 | 0.015475574 |
| MIR762HG | -0.507695166 | 0.01565123 |
| ARHGEF35-AS1 | -0.97820618 | 0.01568795 |
| CACNA1C-AS2 | -0.932723825 | 0.015732914 |
| LINC00654 | 0.780017963 | 0.016247473 |
| LINC01001 | 0.516766893 | 0.016488183 |
| HCG18 | -0.312049962 | 0.016543078 |
| PTCSC1 | 0.803672575 | 0.016839428 |
| TRG-AS1 | -0.493958569 | 0.017775585 |
| LINC00665 | -0.829292802 | 0.019037885 |
| ECE1-AS1 | 0.58989243 | 0.019235979 |
| CKMT2-AS1 | -0.657436557 | 0.019538732 |
| SLC22A18AS | 0.859704174 | 0.020051628 |
| LINC01191 | 0.755859696 | 0.020181162 |
| EIF1B-AS1 | 0.71047079 | 0.020600308 |
| PCCA-DT | -1.092841003 | 0.021315676 |
| GAU1 | 0.983335047 | 0.02147737 |
| LINC01431 | -0.866963775 | 0.021936031 |
| LINC02213 | 1.088298688 | 0.022343835 |
| GUSBP11 | -0.259222093 | 0.022472436 |
| NCF4-AS1 | 0.57630629 | 0.022695734 |
| FAM106C | -1.147086029 | 0.023706369 |
| LINC02860 | 1.182971936 | 0.023973682 |
| MKNK1-AS1 | 0.713744133 | 0.024452404 |
| LINC01160 | 0.632130975 | 0.02449836 |
| CLIP1-AS1 | 1.132674383 | 0.024843991 |
| LINC01189 | -1.254959093 | 0.025084126 |
| SNHG7 | -0.308583647 | 0.025159635 |
| LINC01347 | 0.411829588 | 0.025219679 |
| FAM30A | -0.490413869 | 0.025374576 |
| LINC02731 | -0.941718609 | 0.02556784 |
| LINC02325 | -0.686296671 | 0.025616345 |
| LINC00539 | -0.68537701 | 0.025943121 |
| DIAPH1-AS1 | 1.023831264 | 0.026037037 |
| MIAT | -0.53932985 | 0.026318199 |
| NALT1 | -0.633190986 | 0.026692502 |
| LINC00930 | 1.377141133 | 0.026974654 |
| PRR29-AS1 | -0.978361785 | 0.027413255 |
| VAV3-AS1 | 0.945593151 | 0.027557555 |
| LINC02287 | 1.150627597 | 0.028976929 |
| LINC02604 | -0.286457635 | 0.029334539 |
| RASSF1-AS1 | -0.517745271 | 0.029754165 |
| LINC01569 | -0.491321886 | 0.031129449 |
| MIR155HG | -0.888397194 | 0.031268823 |
| ZNF32-AS2 | -1.00943759 | 0.03156654 |
| LINC00265 | 0.299610636 | 0.031982061 |
| DPP9-AS1 | -0.285979791 | 0.032041228 |
| LINC02397 | -0.620138465 | 0.032059632 |
| ZNF426-DT | -0.959997528 | 0.0321643 |
| LINC02615 | -0.898123478 | 0.032167971 |
| WARS2-AS1 | -0.487721641 | 0.0321952 |
| LINC01772 | -0.464070206 | 0.032811043 |
| MIR3142HG | -0.673161504 | 0.033461649 |
| HAR1A | -0.576299554 | 0.034120982 |
| PITRM1-AS1 | -0.504560131 | 0.035268643 |
| UGDH-AS1 | -0.811480888 | 0.035298708 |
| BFSP2-AS1 | -0.878038722 | 0.03570474 |
| LINC02470 | 1.127680803 | 0.036145431 |
| NORAD | -0.244338356 | 0.036351936 |
| IQCH-AS1 | -0.519278478 | 0.036361482 |
| C1orf220 | -0.668094792 | 0.036412079 |
| TET2-AS1 | 0.939485027 | 0.036612978 |
| LINC02762 | -0.760684389 | 0.03667989 |
| RNF157-AS1 | -0.864087573 | 0.036856974 |
| GABPB1-IT1 | -0.388462723 | 0.036976119 |
| LINC01134 | 0.729578594 | 0.037370768 |
| LINC00638 | -0.732686887 | 0.037722733 |
| C2-AS1 | 1.290171759 | 0.037761945 |
| CSNK1G2-AS1 | 0.592891219 | 0.038576112 |
| MIF-AS1 | -0.869691998 | 0.039133415 |
| PEG13 | -0.561994441 | 0.039949409 |
| PRR34-AS1 | 0.444631189 | 0.040068522 |
| RNF216P1 | -0.175385132 | 0.040191846 |
| LINC01685 | -1.290224149 | 0.041429507 |
| CLRN1-AS1 | 1.269123257 | 0.042119674 |
| DPYD-AS1 | 1.108399604 | 0.042127633 |
| TRHDE-AS1 | 1.273275985 | 0.042441902 |
| AFAP1-AS1 | 1.164296067 | 0.042592438 |
| LINC01694 | -0.960490744 | 0.04265799 |
| MGC4859 | 0.544792874 | 0.043216632 |
| MALINC1 | -0.735366194 | 0.04322796 |
| ALMS1-IT1 | -0.838253791 | 0.043286638 |
| LINC00885 | 1.114898213 | 0.0436848 |
| INKA2-AS1 | 0.897576519 | 0.04377416 |
| ZSWIM8-AS1 | 0.414933624 | 0.04433978 |
| CHN2-AS1 | 0.990360263 | 0.044580037 |
| C1QTNF7-AS1 | 0.964196565 | 0.04494172 |
| ASB16-AS1 | -0.232149017 | 0.045420647 |
| DGCR5 | 0.69235529 | 0.045423128 |
| FAM66D | -1.109262714 | 0.045743285 |
| EDNRB-AS1 | -1.071958868 | 0.04592974 |
| LMNTD2-AS1 | -0.496793604 | 0.045994715 |
| JMJD1C-AS1 | 0.926963765 | 0.046213742 |
| LINC01556 | 1.261351023 | 0.048131555 |
| LINC00467 | 0.596539164 | 0.048530129 |

| **SARS-CoV2 versus healthy control** | | |
| --- | --- | --- |
| **geneID** | **Log2FoldChange** | **Padj** |
| HCP5 | 6.991223779 | 8.32E-101 |
| NEAT1 | 8.437143872 | 1.01E-93 |
| SPATA13 | 5.991427504 | 3.19E-90 |
| DLEU2 | 6.637194055 | 1.02E-87 |
| SNHG6 | 6.896312966 | 6.55E-83 |
| EBLN3P | 7.569242185 | 7.51E-83 |
| PSMB8-AS1 | 8.487349661 | 8.99E-71 |
| NUTM2A-AS1 | 7.14674111 | 5.37E-64 |
| DOCK8-AS1 | 8.49235329 | 6.96E-55 |
| MIR4435-2HG | 6.407988481 | 9.42E-55 |
| CARD8-AS1 | 6.458230219 | 4.21E-52 |
| PCBP1-AS1 | 8.172748033 | 2.65E-47 |
| SNHG29 | 5.534462537 | 4.80E-47 |
| LINC00667 | 6.162023547 | 2.37E-41 |
| CYTOR | 5.951429222 | 1.35E-40 |
| PATL2 | 7.915064865 | 1.52E-38 |
| LINC01506 | 10.65458665 | 1.26E-37 |
| CARINH | 9.147999751 | 1.68E-37 |
| HCG18 | 5.854786564 | 3.03E-37 |
| GABPB1-AS1 | 7.960337821 | 8.95E-37 |
| LINC00877 | 7.435796988 | 8.14E-36 |
| HCG27 | 9.986067176 | 2.74E-35 |
| LINC01410 | 9.525388455 | 1.03E-31 |
| LINC00861 | 9.351219798 | 2.88E-31 |
| RARA-AS1 | 7.850042296 | 3.75E-30 |
| LINC02352 | 9.137351459 | 4.47E-30 |
| PCED1B-AS1 | 8.390181466 | 5.85E-30 |
| ITPK1-AS1 | 8.153066051 | 8.51E-30 |
| LINC00570 | 9.469649451 | 1.62E-29 |
| CFLAR-AS1 | 7.421081822 | 1.99E-28 |
| SNHG1 | 4.675346095 | 9.95E-28 |
| CFAP58-DT | 7.263407459 | 3.01E-26 |
| TRG-AS1 | 9.101291544 | 9.62E-26 |
| LINC02422 | 8.505749186 | 3.02E-25 |
| LINC01138 | 8.827225286 | 5.87E-25 |
| SNHG8 | 6.258228009 | 2.96E-24 |
| JPX | 7.53784628 | 1.83E-23 |
| LINC00342 | 7.043347431 | 6.24E-23 |
| HOTAIRM1 | 8.548779113 | 7.90E-23 |
| PRKCQ-AS1 | 8.718126541 | 9.98E-23 |
| GSEC | 8.937477953 | 3.37E-22 |
| LINC01094 | 5.538373254 | 9.07E-22 |
| GAS5 | 3.56347064 | 9.74E-22 |
| EPB41L4A-AS1 | 5.540866622 | 1.26E-21 |
| RERE-AS1 | 8.004677022 | 5.21E-21 |
| LINC00528 | 7.513209439 | 6.65E-21 |
| DHRS4-AS1 | 7.555328813 | 1.48E-20 |
| SNHG14 | 5.890488113 | 3.35E-20 |
| CHROMR | 5.764146632 | 4.57E-20 |
| DANCR | 5.550740208 | 1.15E-19 |
| PIK3CD-AS1 | 7.367859411 | 2.34E-19 |
| EIF1B-AS1 | 7.15556629 | 9.94E-19 |
| MHENCR | 7.356063399 | 1.12E-18 |
| RAD51-AS1 | 6.850518434 | 1.97E-18 |
| BISPR | 6.307590031 | 2.60E-18 |
| ADAMTSL4-AS1 | 7.433178679 | 2.68E-18 |
| ILF3-DT | 7.140828565 | 2.76E-18 |
| SCARNA9 | 7.344890723 | 6.56E-18 |
| FAM157C | 7.546571948 | 1.81E-17 |
| HEIH | 3.845083169 | 2.89E-17 |
| LINC00623 | 7.106436808 | 3.91E-17 |
| GUSBP11 | 7.085515892 | 1.78E-16 |
| LINC01184 | 6.052917238 | 2.03E-16 |
| GSN-AS1 | 7.511763197 | 2.38E-16 |
| TSPOAP1-AS1 | 6.814189082 | 7.62E-16 |
| LINC01128 | 4.909803509 | 8.34E-16 |
| PSMD6-AS2 | 7.248694665 | 9.83E-16 |
| LINC01278 | 6.945804986 | 1.04E-15 |
| LINC01270 | 6.509069396 | 1.27E-15 |
| NRIR | 7.498081698 | 1.73E-15 |
| LINC00205 | 6.761199871 | 3.21E-15 |
| ATP11A-AS1 | 7.326741092 | 6.49E-15 |
| LINC00649 | 7.480379175 | 7.26E-15 |
| LINC01531 | 7.326053979 | 7.38E-15 |
| FAM30A | 8.157880002 | 7.64E-15 |
| LINC02217 | 7.512496152 | 1.18E-14 |
| LINC02649 | 6.730518857 | 2.27E-14 |
| HIF1A-AS3 | 6.69569797 | 2.54E-14 |
| LINC01002 | 6.721191954 | 4.93E-14 |
| CCR5AS | 4.795773052 | 5.77E-14 |
| HLA-F-AS1 | 6.537979199 | 6.05E-14 |
| SNHG32 | 5.513267138 | 6.19E-14 |
| SENCR | 5.749988004 | 2.24E-13 |
| A2M-AS1 | 7.542491086 | 2.93E-13 |
| KCNJ2-AS1 | 5.983999618 | 3.61E-13 |
| RNF213-AS1 | 5.724643744 | 7.94E-13 |
| LINC02471 | 6.980684831 | 1.64E-12 |
| LINC02656 | 6.386279844 | 3.45E-12 |
| LINC01089 | 6.206223457 | 5.79E-12 |
| LINC00954 | 5.804282118 | 6.38E-12 |
| LINC02273 | 6.921712797 | 7.78E-12 |
| SNHG17 | 4.59284377 | 1.03E-11 |
| LINC01215 | 6.531663448 | 2.79E-11 |
| LINC01176 | 6.68017363 | 4.68E-11 |
| CTBP1-DT | 5.703888703 | 1.01E-10 |
| LINC01550 | 7.229380951 | 1.95E-10 |
| SNHG7 | 4.29173494 | 2.80E-10 |
| LINC02446 | 7.058745406 | 1.23E-09 |
| LINC01762 | 5.628657154 | 2.01E-09 |
| LINC00685 | 5.458462748 | 4.21E-09 |
| IL10RB-DT | 5.177291328 | 4.90E-09 |
| MIR3945HG | 3.061091852 | 6.52E-09 |
| LINC00926 | 6.245096013 | 7.41E-09 |
| LINC02611 | 6.165234951 | 9.15E-09 |
| LINC01093 | 6.544470986 | 1.41E-08 |
| LINC01588 | 2.832171198 | 1.89E-08 |
| CKMT2-AS1 | 5.480589644 | 2.05E-08 |
| LINC00482 | 5.49489451 | 4.69E-08 |
| LINC01303 | 4.340574033 | 6.19E-08 |
| LINC01353 | 5.345658502 | 6.92E-08 |
| LINC02363 | 4.893936598 | 1.67E-07 |
| TAPT1-AS1 | 5.411632717 | 2.20E-07 |
| SATB1-AS1 | 5.111422811 | 2.68E-07 |
| VIPR1-AS1 | 4.846688395 | 7.87E-07 |
| LINC02397 | 6.093135024 | 1.31E-06 |
| MAP3K5-AS1 | 4.90124403 | 1.80E-06 |
| LINC01485 | 5.250048933 | 2.56E-06 |
| BASP1-AS1 | 4.463531464 | 3.23E-06 |
| SLC25A25-AS1 | 4.180850111 | 8.92E-06 |
| LINC01191 | 4.648192395 | 1.51E-05 |
| DISC1-IT1 | 5.915759192 | 1.52E-05 |
| LINC00663 | 4.975425988 | 2.16E-05 |
| MDS2 | 5.242690423 | 2.58E-05 |
| LINC00654 | 4.071002529 | 4.09E-05 |
| LINC01006 | 3.95647245 | 0.00010924 |
| LINC02325 | 4.718401868 | 0.00017022 |
| LINC00526 | 4.506756885 | 0.00021505 |
| PKN2-AS1 | 3.336412609 | 0.00037938 |
| C5orf67 | 4.501804779 | 0.00038746 |
| AOAH-IT1 | 4.254588655 | 0.00040162 |
| LINC02648 | 4.266042109 | 0.00078904 |
| KLF3-AS1 | 2.961976229 | 0.00080662 |
| MKNK1-AS1 | 3.980678741 | 0.0073359 |
| HOXB-AS1 | 3.020895531 | 0.0104613 |
| PITRM1-AS1 | 3.637364294 | 0.0165923 |
| LINC02528 | 3.771918948 | 0.017681 |
| CEROX1 | 3.557041274 | 0.02926378 |
| LUCAT1 | 6.053486683 | 1.39E-172 |
| NORAD | 5.995646674 | 3.45E-149 |
| MIR223HG | 10.23093077 | 2.00E-148 |
| ZFAS1 | 6.570878452 | 1.63E-146 |
| MALAT1 | 6.938262793 | 3.65E-129 |
| FTX | 8.013136535 | 1.38E-123 |
| PSMA3-AS1 | 7.282962392 | 1.51E-108 |
| VIM-AS1 | 6.684639102 | 1.40E-80 |
| THUMPD3-AS1 | 6.124317838 | 1.41E-76 |
| GARS1-DT | 7.683462892 | 3.67E-74 |
| SNHG5 | 6.471734169 | 1.14E-72 |
| FGD5-AS1 | 5.80700692 | 7.63E-72 |
| OIP5-AS1 | 4.627469808 | 8.34E-69 |
| LINC-PINT | 6.270613141 | 1.03E-68 |
| WAC-AS1 | 7.320979431 | 4.55E-57 |
| LRRK2-DT | 10.18326715 | 6.39E-54 |
| ENTPD1-AS1 | 6.574568765 | 1.14E-52 |
| CCDC18-AS1 | 7.752059403 | 5.26E-47 |
| GABPB1-IT1 | 7.143951343 | 2.84E-46 |
| SMIM10L1 | 5.998476426 | 3.50E-46 |
| ZNF84-DT | -10.93936898 | 3.52E-45 |
| KCNQ1OT1 | 5.882390787 | 3.94E-45 |
| OTUD6B-AS1 | 5.733274066 | 1.58E-43 |
| NUTM2B-AS1 | 7.286554381 | 9.81E-41 |
| RAB30-DT | 6.598594414 | 2.58E-38 |
| LINC00963 | 6.073655622 | 4.91E-38 |
| LINC00324 | 6.678680183 | 5.05E-38 |
| ALOX12-AS1 | 7.720740342 | 1.09E-36 |
| MIR29B2CHG | 7.640556333 | 1.61E-34 |
| LINC01003 | 6.590617283 | 2.48E-32 |
| LINC02772 | 10.35038169 | 2.73E-32 |
| DLEU1 | 9.804721686 | 1.06E-31 |
| TTN-AS1 | 9.025350733 | 1.46E-31 |
| NFE4 | 10.01533023 | 9.35E-31 |
| NPTN-IT1 | 6.554571684 | 2.80E-30 |
| ANKRD44-IT1 | 9.768132002 | 2.89E-30 |
| TPT1-AS1 | 4.406668871 | 2.92E-30 |
| SLC8A1-AS1 | 9.612535588 | 3.17E-30 |
| DNAJC3-DT | 7.190119939 | 3.47E-30 |
| FAM13A-AS1 | 9.188303646 | 1.08E-29 |
| OSER1-DT | 6.478785371 | 3.32E-29 |
| SAP30L-AS1 | 8.938511476 | 1.32E-28 |
| MIAT | 5.846478536 | 3.63E-28 |
| SNHG10 | 8.895823813 | 4.49E-28 |
| TMEM44-AS1 | 5.5314514 | 3.05E-27 |
| BOLA3-AS1 | 8.824596381 | 3.59E-27 |
| APTR | 8.748141373 | 3.84E-27 |
| ITGB2-AS1 | 8.673547133 | 6.19E-27 |
| KDM7A-DT | 8.007932665 | 1.88E-26 |
| LINC02555 | 9.267120599 | 2.40E-26 |
| NINJ2-AS1 | 8.68748586 | 4.40E-26 |
| MIRLET7A1HG | 5.402798771 | 1.48E-25 |
| LINC00641 | 6.101832494 | 1.72E-25 |
| ZBED5-AS1 | 6.086358454 | 3.77E-25 |
| INTS6-AS1 | 8.420758939 | 7.45E-25 |
| PPM1F-AS1 | 8.423491735 | 2.25E-24 |
| LINC02035 | 7.688814901 | 2.42E-24 |
| NUP50-DT | 6.968844009 | 2.65E-24 |
| PLBD1-AS1 | 8.613201126 | 4.12E-24 |
| MUC20-OT1 | 5.555576532 | 1.01E-23 |
| LINC00662 | 6.594347798 | 1.12E-23 |
| MIATNB | 7.211494949 | 2.43E-23 |
| ABALON | 8.564005463 | 2.78E-23 |
| SH3BP5-AS1 | 8.085160258 | 3.45E-23 |
| CELF2-AS1 | 8.626994412 | 4.36E-23 |
| NIPBL-DT | 6.80112407 | 4.57E-23 |
| LINC02207 | 9.289330399 | 5.29E-23 |
| CD27-AS1 | 7.983982036 | 6.43E-23 |
| PRR34-AS1 | 7.962889604 | 1.35E-22 |
| ASAP1-IT2 | 8.085140079 | 1.42E-22 |
| LINC02863 | 8.435394656 | 1.75E-22 |
| LINC01127 | 8.853293289 | 2.06E-22 |
| N4BP2L2-IT2 | 7.926038076 | 2.12E-22 |
| LINC01765 | 9.028687667 | 2.18E-22 |
| MBNL1-AS1 | 8.165917462 | 2.39E-22 |
| NIFK-AS1 | 6.808657953 | 3.30E-22 |
| LINC00211 | 8.936896573 | 3.80E-22 |
| RTCA-AS1 | 8.278411152 | 4.05E-22 |
| GIHCG | 7.937703053 | 4.13E-22 |
| SNHG16 | 6.078051119 | 4.19E-22 |
| MAPKAPK5-AS1 | 6.982126097 | 1.10E-21 |
| ASB16-AS1 | 7.641448654 | 1.56E-21 |
| MMP25-AS1 | 7.69419268 | 2.11E-21 |
| SEPSECS-AS1 | 8.084858931 | 4.24E-21 |
| GMDS-DT | 6.842579297 | 8.34E-21 |
| LINC00173 | 7.689050808 | 1.91E-20 |
| TRIM52-AS1 | 7.909829764 | 2.66E-20 |
| PTPRJ-AS1 | 8.146456314 | 3.68E-20 |
| MIR646HG | 7.898589741 | 4.79E-20 |
| LINC00989 | 8.299716049 | 5.28E-20 |
| CA3-AS1 | 8.927377086 | 8.26E-20 |
| LINC00892 | 8.214707809 | 9.18E-20 |
| TTTY8 | -7.878595513 | 1.64E-19 |
| LGALSL-DT | 7.633972947 | 3.89E-19 |
| C8orf44 | 7.566661001 | 8.81E-19 |
| LINC02288 | 8.481825566 | 1.19E-18 |
| EIF3J-DT | 6.735622162 | 1.38E-18 |
| LINC00937 | 8.064360605 | 1.70E-18 |
| LINC00921 | 7.506768658 | 1.91E-18 |
| LINC02213 | 7.324070051 | 2.36E-18 |
| DLGAP1-AS1 | 6.826976283 | 3.37E-18 |
| SNHG20 | 6.639153195 | 3.71E-18 |
| LINC02751 | 7.967553026 | 3.77E-18 |
| PRANCR | 7.058584204 | 4.08E-18 |
| DAXX | 1.813329598 | 4.53E-18 |
| SNHG3 | 5.545605341 | 4.84E-18 |
| SDCBP2-AS1 | 7.048008676 | 5.86E-18 |
| LINC02785 | 6.77026164 | 9.01E-18 |
| NDUFV2-AS1 | 7.108709171 | 1.08E-17 |
| LINC02158 | 7.531695567 | 1.32E-17 |
| MRPL20-AS1 | 7.264394947 | 1.49E-17 |
| TTC28-AS1 | 7.256086472 | 1.95E-17 |
| LINC02762 | 7.063576476 | 2.32E-17 |
| NBR2 | 6.980202473 | 2.76E-17 |
| MAPK4 | -7.607663646 | 2.84E-17 |
| LINC00476 | 7.87615297 | 3.06E-17 |
| PLCG1-AS1 | 7.251154575 | 3.19E-17 |
| LINC01355 | 7.345998151 | 3.58E-17 |
| MKLN1-AS | 6.835408565 | 4.06E-17 |
| STXBP5-AS1 | 5.354224218 | 4.83E-17 |
| LINC02289 | 8.071967769 | 5.32E-17 |
| ZNF213-AS1 | 6.838068458 | 6.35E-17 |
| TP53TG1 | 4.680506519 | 6.96E-17 |
| KANSL1-AS1 | 8.182690143 | 7.93E-17 |
| TET2-AS1 | 7.206971376 | 8.72E-17 |
| PPP3CB-AS1 | 5.681221801 | 1.05E-16 |
| SNHG30 | 6.936006324 | 1.26E-16 |
| THAP9-AS1 | 3.979119764 | 1.29E-16 |
| RUSC1-AS1 | 6.892926532 | 1.76E-16 |
| ZSCAN16-AS1 | 6.785267523 | 1.96E-16 |
| LNCAROD | 6.983359852 | 2.31E-16 |
| LINC01684 | 7.57152818 | 2.63E-16 |
| NCK1-DT | 6.600510647 | 2.71E-16 |
| GK-IT1 | 7.262551221 | 3.35E-16 |
| LINC02384 | 7.30624403 | 3.51E-16 |
| SND1-IT1 | 6.843378551 | 3.53E-16 |
| LINC02680 | 6.578042814 | 3.62E-16 |
| LINC00513 | 6.788390387 | 3.74E-16 |
| ERVK13-1 | 6.15725888 | 7.13E-16 |
| LINC01504 | 7.16788852 | 7.32E-16 |
| LINC00910 | 6.979802728 | 7.63E-16 |
| FOXP1-AS1 | 6.891999459 | 9.32E-16 |
| ARHGAP15-AS1 | 7.133249938 | 1.03E-15 |
| LINC00534 | 7.39630665 | 1.04E-15 |
| OVCH1-AS1 | -2.627891844 | 1.08E-15 |
| VPS13B-DT | 7.237953934 | 1.09E-15 |
| CPB2-AS1 | 6.704362576 | 1.17E-15 |
| LINC00243 | 7.123934986 | 1.74E-15 |
| LRRC8C-DT | 5.05381833 | 2.29E-15 |
| LINC01948 | 7.300805247 | 2.36E-15 |
| USP3-AS1 | 7.19507111 | 2.78E-15 |
| UBE2D3-AS1 | 6.801033868 | 3.70E-15 |
| FOXP1-IT1 | 7.254074112 | 3.92E-15 |
| SBF2-AS1 | 6.843841949 | 4.44E-15 |
| DIAPH1-AS1 | 7.195350457 | 4.65E-15 |
| ASH1L-AS1 | 6.681103114 | 4.73E-15 |
| POC1B-AS1 | 6.556213785 | 5.02E-15 |
| PDCD4-AS1 | 6.893326562 | 5.87E-15 |
| ARRDC1-AS1 | 6.309881625 | 5.94E-15 |
| SREBF2-AS1 | 6.385225418 | 6.50E-15 |
| LINC01232 | 7.303600114 | 6.77E-15 |
| LINC02362 | 6.240028988 | 7.35E-15 |
| RASA2-IT1 | 6.738313168 | 9.62E-15 |
| DRAIC | 7.021405718 | 9.99E-15 |
| ZEB1-AS1 | 6.748401348 | 1.00E-14 |
| NNT-AS1 | 6.075641038 | 1.66E-14 |
| SCAT8 | 6.878262423 | 1.67E-14 |
| SNHG15 | 3.462962816 | 1.70E-14 |
| ST20-AS1 | 6.482648947 | 3.19E-14 |
| SLC16A1-AS1 | 6.507715828 | 3.46E-14 |
| LINC00467 | 6.448596424 | 5.56E-14 |
| PAN3-AS1 | 7.000878776 | 5.60E-14 |
| FLVCR1-DT | 6.81940209 | 7.04E-14 |
| ANKRD10-IT1 | 6.755297327 | 8.02E-14 |
| SNHG26 | 7.12869161 | 8.89E-14 |
| LINC01359 | 6.818359937 | 9.30E-14 |
| SNHG9 | 6.733741704 | 9.50E-14 |
| COX10-AS1 | 7.032153367 | 1.06E-13 |
| LINC00299 | 6.781984941 | 1.09E-13 |
| ARHGAP26-IT1 | 7.039384614 | 1.19E-13 |
| LINC00671 | 6.713064726 | 1.20E-13 |
| RNF216P1 | 5.696329365 | 1.36E-13 |
| SCAMP1-AS1 | 5.931457042 | 1.52E-13 |
| GHRLOS | 6.465638844 | 1.67E-13 |
| LINC01011 | 6.923832814 | 1.77E-13 |
| LINC02432 | 6.945510653 | 2.14E-13 |
| LINC00539 | 6.893664416 | 2.33E-13 |
| CHRM3-AS2 | 7.292575186 | 2.46E-13 |
| LINC00294 | 5.96018583 | 2.74E-13 |
| VCAN-AS1 | 6.394670287 | 3.40E-13 |
| PDC-AS1 | 7.102968098 | 3.45E-13 |
| WDFY3-AS2 | 5.072920276 | 3.48E-13 |
| DPYD-AS2 | 6.894921654 | 4.27E-13 |
| B4GALT1-AS1 | 6.923526232 | 4.40E-13 |
| SNAI3-AS1 | 6.381507931 | 4.72E-13 |
| MIR181A1HG | 4.466892063 | 5.03E-13 |
| MIR133A1HG | 6.65175336 | 5.11E-13 |
| SNHG12 | 5.820157524 | 5.42E-13 |
| C1RL-AS1 | 6.022034944 | 6.98E-13 |
| LINC00862 | 6.169037761 | 7.59E-13 |
| ITCH-IT1 | 6.622425276 | 9.54E-13 |
| ITGA6-AS1 | 6.894995819 | 9.72E-13 |
| LINC00847 | 6.668944663 | 9.80E-13 |
| RELA-DT | 6.311237678 | 9.80E-13 |
| ZFHX2-AS1 | 6.112922495 | 1.20E-12 |
| LINC01503 | 6.166057996 | 1.34E-12 |
| LINC00639 | 6.36749308 | 1.35E-12 |
| LINC02580 | 6.536710688 | 1.43E-12 |
| WAKMAR2 | 6.474252433 | 1.44E-12 |
| AATBC | 6.387844305 | 1.47E-12 |
| ZNNT1 | 6.617357197 | 1.74E-12 |
| DICER1-AS1 | 6.514865598 | 2.48E-12 |
| PVT1 | 5.65087607 | 2.51E-12 |
| LINC00853 | 6.282242262 | 2.79E-12 |
| LINC01934 | 6.587799516 | 3.46E-12 |
| ANKRD34C-AS1 | -6.921693495 | 3.53E-12 |
| MANEA-DT | 7.345010596 | 4.19E-12 |
| PANK2-AS1 | 5.938934939 | 4.19E-12 |
| CYP1B1-AS1 | 6.219285359 | 4.23E-12 |
| ENTPD3-AS1 | 6.004251744 | 4.86E-12 |
| ECE1-AS1 | 6.430128137 | 5.13E-12 |
| MCPH1-AS1 | 6.279033899 | 5.92E-12 |
| MAP3K2-DT | 5.617031381 | 6.11E-12 |
| MRTFA-AS1 | 6.487682512 | 6.72E-12 |
| LINC00899 | 5.948065382 | 6.97E-12 |
| MIRLET7BHG | 6.090583997 | 8.07E-12 |
| FAM111A-DT | 6.445506897 | 1.00E-11 |
| LINC00426 | 6.364469116 | 1.25E-11 |
| PAXIP1-AS2 | 6.088399293 | 1.36E-11 |
| TMPO-AS1 | 6.664140548 | 1.96E-11 |
| LINC02019 | 6.011771753 | 2.09E-11 |
| AIRN | 6.601115648 | 2.30E-11 |
| KANSL1L-AS1 | 6.403455226 | 2.35E-11 |
| HCG25 | 6.352560454 | 2.35E-11 |
| NOP14-AS1 | 5.676895605 | 3.03E-11 |
| TNK2-AS1 | 6.00410496 | 3.07E-11 |
| LINC01963 | 6.07042041 | 3.42E-11 |
| ATP2B1-AS1 | 2.764555131 | 3.42E-11 |
| RAP2C-AS1 | 6.319667018 | 3.47E-11 |
| ZNF790-AS1 | 6.37835385 | 3.52E-11 |
| PINK1-AS | 5.922183482 | 3.66E-11 |
| ITPKB-IT1 | 5.900994396 | 3.88E-11 |
| LINC01151 | 6.497923115 | 7.14E-11 |
| MIR4453HG | 6.162136024 | 7.20E-11 |
| LINC02328 | 6.559566075 | 7.38E-11 |
| LIPE-AS1 | 5.789224853 | 7.71E-11 |
| ZNF710-AS1 | 5.677170316 | 9.60E-11 |
| MCM3AP-AS1 | 6.743510657 | 9.89E-11 |
| MED14OS | 5.249413702 | 1.11E-10 |
| ATP1A1-AS1 | 6.242255032 | 1.15E-10 |
| ZKSCAN2-DT | 6.147762448 | 1.21E-10 |
| STX18-AS1 | 6.029191077 | 1.24E-10 |
| LINC02284 | 5.928785905 | 1.44E-10 |
| ELF3-AS1 | 5.659008526 | 1.77E-10 |
| SNRK-AS1 | 5.932892628 | 1.87E-10 |
| STIM2-AS1 | 6.491503764 | 1.91E-10 |
| CHKB-DT | 5.643692199 | 1.97E-10 |
| ELOA-AS1 | 6.034020446 | 2.08E-10 |
| LINC01871 | 6.792081346 | 2.12E-10 |
| LINC02723 | 5.828357153 | 2.21E-10 |
| BTBD9-AS1 | 5.760666488 | 2.37E-10 |
| DIAPH2-AS1 | 5.636305617 | 2.51E-10 |
| TNFRSF14-AS1 | 5.83322574 | 2.71E-10 |
| LINC00894 | 5.911263701 | 2.84E-10 |
| DPYD-AS1 | 6.36189059 | 3.08E-10 |
| LINC01772 | 6.118789373 | 3.16E-10 |
| RFX3-AS1 | 6.192140682 | 3.26E-10 |
| ENO1-AS1 | 6.083200915 | 3.43E-10 |
| PAXBP1-AS1 | 5.560764343 | 3.76E-10 |
| LINC02256 | 5.71524932 | 3.77E-10 |
| CEP250-AS1 | 5.828170366 | 4.08E-10 |
| LINC02211 | -6.99082959 | 4.50E-10 |
| STAG3L5P-PVRIG2P-PILRB | 5.645308557 | 5.57E-10 |
| DGUOK-AS1 | 5.833665429 | 5.82E-10 |
| BAIAP2-DT | 6.370467315 | 6.21E-10 |
| INE1 | 5.700097777 | 6.29E-10 |
| ZNF561-AS1 | 5.899621781 | 7.22E-10 |
| E2F3-IT1 | 6.159539695 | 7.26E-10 |
| URB1-AS1 | 5.605715687 | 7.39E-10 |
| SGMS1-AS1 | 5.434065616 | 7.67E-10 |
| CAPN10-DT | 5.455467917 | 7.68E-10 |
| LINC02631 | 5.763090055 | 8.98E-10 |
| ITPRIP-AS1 | 5.703668602 | 9.46E-10 |
| LUNAR1 | 6.277267903 | 1.19E-09 |
| RORA-AS1 | 5.828147264 | 1.34E-09 |
| LINC00863 | 6.030130078 | 1.45E-09 |
| KIF9-AS1 | 5.612810526 | 1.57E-09 |
| TPRG1-AS1 | 5.887839519 | 1.58E-09 |
| PRR7-AS1 | 6.07709963 | 1.61E-09 |
| STARD7-AS1 | 5.436738823 | 1.63E-09 |
| LINC02803 | 6.110223957 | 1.90E-09 |
| BACE1-AS | 6.143301508 | 2.09E-09 |
| MINCR | 6.239319231 | 2.32E-09 |
| UVRAG-DT | 5.983061635 | 2.44E-09 |
| LOXL1-AS1 | 5.958493784 | 2.49E-09 |
| LINC02812 | 6.080533545 | 2.68E-09 |
| LPP-AS2 | 5.694937777 | 2.80E-09 |
| SNHG11 | 5.56551257 | 3.07E-09 |
| LINC00265 | 5.901630894 | 3.09E-09 |
| HIPK1-AS1 | 5.900314505 | 3.45E-09 |
| LINC01781 | 6.432232513 | 3.59E-09 |
| LINC02018 | 5.656219746 | 4.35E-09 |
| PXN-AS1 | 5.573063328 | 4.56E-09 |
| LINC01137 | 5.412616377 | 4.85E-09 |
| LINC00339 | 5.23502181 | 5.60E-09 |
| WARS2-AS1 | 5.556447549 | 5.69E-09 |
| LINC02245 | 5.968229722 | 5.97E-09 |
| LINC00239 | 5.748554149 | 6.04E-09 |
| DHDDS-AS1 | 5.930565989 | 7.01E-09 |
| LINC01534 | 5.92159085 | 7.16E-09 |
| BHLHE40-AS1 | 5.716338903 | 7.21E-09 |
| LINC01560 | 5.811316417 | 7.35E-09 |
| LINC01513 | 5.561302296 | 7.83E-09 |
| C21orf91-OT1 | 5.248216916 | 7.93E-09 |
| ADAMTSL4-AS2 | 5.432498838 | 7.96E-09 |
| LINC01146 | 5.651430844 | 8.44E-09 |
| ARMCX5-GPRASP2 | 5.825932786 | 8.72E-09 |
| ZNF337-AS1 | 5.836786006 | 8.88E-09 |
| ZNF529-AS1 | 5.083282417 | 9.61E-09 |
| EPHA1-AS1 | 6.072319103 | 9.72E-09 |
| MIR22HG | 2.081705108 | 9.90E-09 |
| LACTB2-AS1 | 5.626535783 | 1.09E-08 |
| LINC02724 | 5.279088389 | 1.25E-08 |
| ZNF674-AS1 | 3.795988318 | 1.25E-08 |
| LINC02175 | 6.168106342 | 1.26E-08 |
| ITGA9-AS1 | 5.404528925 | 1.26E-08 |
| LINC02481 | 6.212811752 | 1.27E-08 |
| ZNF451-AS1 | 6.075648439 | 1.29E-08 |
| SEMA3F-AS1 | 5.154441312 | 1.46E-08 |
| ARAP1-AS2 | 5.645185477 | 1.78E-08 |
| TRHDE-AS1 | 5.700458775 | 2.23E-08 |
| SCAT2 | 4.354421565 | 2.35E-08 |
| WDR86-AS1 | 6.119720124 | 2.38E-08 |
| SP2-AS1 | 5.481754099 | 2.49E-08 |
| ZNF687-AS1 | 5.459533342 | 3.19E-08 |
| NSMCE1-DT | 5.4593605 | 3.46E-08 |
| LINC02340 | 5.095469451 | 3.64E-08 |
| IQCH-AS1 | 5.172265659 | 3.92E-08 |
| HDAC4-AS1 | 5.543032805 | 4.01E-08 |
| ANKRD44-AS1 | 5.62505717 | 4.03E-08 |
| ZNF236-DT | 5.290003832 | 6.64E-08 |
| LINC02470 | 8.258063164 | 6.67E-08 |
| LINC00630 | 5.4935974 | 6.78E-08 |
| LINC02009 | 6.900319661 | 8.69E-08 |
| NFYC-AS1 | 5.745525491 | 8.72E-08 |
| LINC01136 | 5.830663216 | 9.95E-08 |
| C21orf62-AS1 | 3.955739841 | 1.02E-07 |
| ZNRD1ASP | 4.788196511 | 1.04E-07 |
| LINC00957 | 5.184655154 | 1.06E-07 |
| LINC01422 | 5.412668009 | 1.09E-07 |
| TMEM9B-AS1 | 5.343858266 | 1.20E-07 |
| GK-AS1 | 5.915264905 | 1.27E-07 |
| SAP30-DT | 5.418194232 | 1.33E-07 |
| ZBTB11-AS1 | 4.977598248 | 1.38E-07 |
| ZNF667-AS1 | 4.760396975 | 1.45E-07 |
| DPYD-IT1 | 5.605792318 | 1.47E-07 |
| FRY-AS1 | 5.156359459 | 1.49E-07 |
| ZNF252P-AS1 | 5.594458423 | 1.54E-07 |
| HHLA3 | 5.02783537 | 1.74E-07 |
| LYPLAL1-DT | 5.758624154 | 1.90E-07 |
| LINC01352 | 5.405094428 | 2.04E-07 |
| CASC19 | 5.83908243 | 2.05E-07 |
| ADNP-AS1 | 5.654375384 | 2.17E-07 |
| LY86-AS1 | 5.712565838 | 2.19E-07 |
| DDN-AS1 | 5.141206747 | 2.46E-07 |
| RNASEH2B-AS1 | 5.279948875 | 2.66E-07 |
| ERICH6-AS1 | 5.038512218 | 2.70E-07 |
| PCAT1 | 5.26953925 | 3.15E-07 |
| KLF7-IT1 | 5.248509319 | 3.52E-07 |
| LINC01637 | 4.832695958 | 3.93E-07 |
| PARP11-AS1 | 6.040408761 | 4.14E-07 |
| LATS2-AS1 | 5.336378714 | 4.19E-07 |
| SLC2A1-AS1 | 5.416600825 | 4.32E-07 |
| SOS1-IT1 | 6.040278496 | 4.34E-07 |
| PSMG3-AS1 | 4.933338853 | 5.34E-07 |
| RNF217-AS1 | 5.667062153 | 5.41E-07 |
| HELLPAR | 2.89803849 | 5.42E-07 |
| TMC3-AS1 | 5.068489114 | 5.54E-07 |
| GYG2-AS1 | -3.869194922 | 5.57E-07 |
| VAC14-AS1 | 5.090212842 | 5.89E-07 |
| XIST | 6.115124108 | 6.33E-07 |
| PRKAR1B-AS1 | 4.877701129 | 6.37E-07 |
| SMILR | 5.84833889 | 6.57E-07 |
| MAP3K14-AS1 | 4.625210043 | 7.53E-07 |
| DGCR11 | 5.146961028 | 7.58E-07 |
| LINC00676 | 5.288922954 | 8.01E-07 |
| HECW2-AS1 | 5.890583041 | 8.03E-07 |
| NAPA-AS1 | 4.990283394 | 8.54E-07 |
| PIK3CD-AS2 | 5.138855804 | 8.81E-07 |
| PAX8-AS1 | 5.137110151 | 8.94E-07 |
| LINC00653 | 5.272894425 | 9.45E-07 |
| LINC00968 | 5.241671501 | 9.58E-07 |
| BACH1-IT1 | 4.999522066 | 1.18E-06 |
| LINC02694 | 5.306404604 | 1.20E-06 |
| MEF2C-AS1 | 4.658203838 | 1.43E-06 |
| TRAM2-AS1 | 4.873923175 | 1.66E-06 |
| WDR11-AS1 | 4.08372847 | 1.85E-06 |
| MALINC1 | 5.288279246 | 1.92E-06 |
| RNU6ATAC35P | 5.665804923 | 1.94E-06 |
| LINC00189 | 5.241469494 | 2.01E-06 |
| L3MBTL2-AS1 | 4.919223079 | 2.40E-06 |
| FAM201A | 4.851187502 | 2.46E-06 |
| SNHG22 | 4.908034275 | 2.63E-06 |
| IFNG-AS1 | 5.612323481 | 2.71E-06 |
| WWC3-AS1 | 5.101517253 | 2.99E-06 |
| C2orf27A | 3.717780822 | 3.05E-06 |
| LINC01888 | 5.309215386 | 3.16E-06 |
| ARRDC3-AS1 | 4.677941792 | 3.25E-06 |
| LINC02458 | 6.080429937 | 3.28E-06 |
| MIR3150BHG | 5.232507301 | 3.37E-06 |
| FAM53B-AS1 | 4.680915682 | 3.40E-06 |
| LINC00115 | 5.174860594 | 3.50E-06 |
| LINC01547 | 4.863570208 | 3.53E-06 |
| CEBPB-AS1 | 5.31631778 | 3.79E-06 |
| SEMA6A-AS1 | 5.249897108 | 4.05E-06 |
| LIX1L-AS1 | 4.991609597 | 4.42E-06 |
| RUNDC3A-AS1 | 5.178165844 | 4.67E-06 |
| ZBTB20-AS1 | 5.095344081 | 4.93E-06 |
| CDK6-AS1 | -4.000108252 | 4.98E-06 |
| USP30-AS1 | 5.1245001 | 5.24E-06 |
| DAPK1-IT1 | 5.595243613 | 6.05E-06 |
| SCOC-AS1 | 4.682311657 | 6.48E-06 |
| FOXN3-AS1 | 4.430714897 | 6.49E-06 |
| LINC02604 | 4.7218656 | 6.52E-06 |
| SNHG4 | 4.957046735 | 6.64E-06 |
| OLMALINC | 4.897805262 | 6.93E-06 |
| ARIH2OS | 4.454769491 | 7.27E-06 |
| MPP7-DT | 5.947140026 | 7.56E-06 |
| TBC1D22A-AS1 | 5.179862012 | 7.67E-06 |
| RABGAP1L-IT1 | 5.490865393 | 8.03E-06 |
| LINC02084 | 5.118284791 | 8.79E-06 |
| LINC00884 | 4.192477278 | 8.79E-06 |
| UBOX5-AS1 | 4.61322947 | 8.85E-06 |
| PRKAG2-AS1 | 4.870314212 | 9.01E-06 |
| SKAP1-AS1 | 4.609595335 | 9.24E-06 |
| LBX2-AS1 | 4.170491315 | 9.63E-06 |
| LINC01943 | 4.222439993 | 9.73E-06 |
| RNASEH1-AS1 | 4.478566153 | 1.11E-05 |
| SPAG5-AS1 | 4.27777066 | 1.12E-05 |
| CDKN2B-AS1 | 4.6490211 | 1.20E-05 |
| PHACTR2-AS1 | 4.923724045 | 1.24E-05 |
| TMED2-DT | 5.012984848 | 1.31E-05 |
| CAHM | 4.422482903 | 1.33E-05 |
| TTTY10 | 5.98922557 | 1.53E-05 |
| LINC02482 | 4.679650017 | 1.53E-05 |
| TRAPPC12-AS1 | 4.633632251 | 1.58E-05 |
| DOCK4-AS1 | 5.038657773 | 1.64E-05 |
| TOB1-AS1 | 4.638263373 | 1.88E-05 |
| LINC01480 | 5.004064276 | 2.07E-05 |
| SBNO1-AS1 | 4.197048034 | 2.10E-05 |
| CD44-AS1 | 3.90012385 | 2.11E-05 |
| SIAH2-AS1 | 4.591898433 | 2.25E-05 |
| PPP1R12A-AS1 | 4.591761308 | 2.25E-05 |
| ACAP2-IT1 | 4.880188605 | 2.31E-05 |
| LINC00106 | 4.402035635 | 2.32E-05 |
| MAGI2-AS3 | 4.251383741 | 2.35E-05 |
| LINC01606 | -5.865284787 | 2.52E-05 |
| HSD11B1-AS1 | 5.098285753 | 2.55E-05 |
| DNAH17-AS1 | 4.299685527 | 2.55E-05 |
| CYB561D2 | 1.008660189 | 2.55E-05 |
| BCDIN3D-AS1 | 5.602535823 | 2.57E-05 |
| DLGAP1-AS2 | 3.273714002 | 3.06E-05 |
| ARF4-AS1 | 4.667993531 | 3.13E-05 |
| C1QTNF7-AS1 | 4.403204193 | 3.41E-05 |
| LINC02316 | 4.794059936 | 3.45E-05 |
| LINC01147 | 4.983560275 | 3.53E-05 |
| RC3H1-IT1 | 4.741866624 | 3.65E-05 |
| LINC01814 | 4.91870366 | 3.89E-05 |
| EIF2AK3-DT | 4.530527894 | 4.11E-05 |
| MAP4K3-DT | 3.425767253 | 4.38E-05 |
| BACH1-IT2 | 4.568344487 | 4.63E-05 |
| LINC01220 | 3.96708267 | 4.73E-05 |
| XACT | 2.291872888 | 5.10E-05 |
| LINC01409 | 4.91757074 | 5.47E-05 |
| NEXN-AS1 | 4.234102363 | 5.48E-05 |
| LINC02193 | 4.793096774 | 5.77E-05 |
| TMEM147-AS1 | 4.766370132 | 5.98E-05 |
| SNHG25 | 4.475177015 | 6.06E-05 |
| MIR503HG | 4.206711062 | 6.32E-05 |
| LINC02669 | 4.936602506 | 6.37E-05 |
| TIPARP-AS1 | 4.38985912 | 6.54E-05 |
| JAZF1-AS1 | 4.84862026 | 6.80E-05 |
| LINC00624 | 4.811745175 | 7.70E-05 |
| LINC00174 | 1.435287547 | 7.88E-05 |
| CCNT2-AS1 | 4.997631478 | 8.64E-05 |
| RHOA-IT1 | 4.824620877 | 8.79E-05 |
| SATB2-AS1 | 5.682416601 | 8.91E-05 |
| TRBV11-2 | 4.844717901 | 9.19E-05 |
| LINC00554 | -6.203170667 | 9.20E-05 |
| MIR302CHG | 4.464974449 | 9.46E-05 |
| MAST4-AS1 | 5.57079347 | 9.48E-05 |
| MIR4458HG | 4.984900532 | 9.58E-05 |
| LINC00944 | 5.065683332 | 9.69E-05 |
| LINC01619 | 4.95018041 | 9.99E-05 |
| ZNF22-AS1 | 4.602205925 | 0.00010005 |
| ZNF528-AS1 | 4.420324814 | 0.00010481 |
| TRAF3IP2-AS1 | 3.818413005 | 0.00010633 |
| TTTY14 | 7.01409722 | 0.00011483 |
| SGO1-AS1 | 4.500911339 | 0.00011494 |
| LINC00943 | 4.991985995 | 0.00012231 |
| SIDT1-AS1 | 5.032087389 | 0.00012478 |
| LINC00683 | 3.969485069 | 0.00013137 |
| PHF1 | -0.799258472 | 0.00013457 |
| LINC02371 | 4.676177062 | 0.00013606 |
| MFF-DT | 4.22971435 | 0.00014038 |
| KIFC1 | -1.654814439 | 0.00014493 |
| HYMAI | 4.370372235 | 0.00015167 |
| LINC00824 | 4.762011816 | 0.00015225 |
| LINC01291 | 5.276914868 | 0.00015832 |
| SUCLG2-AS1 | 5.016464941 | 0.00016956 |
| RRN3P2 | 3.657306844 | 0.00018232 |
| MIR924HG | 4.997163205 | 0.00018647 |
| RABGAP1L-DT | 3.950011279 | 0.00018883 |
| LINC01572 | 4.450782021 | 0.00019991 |
| MPRIP-AS1 | 4.378140507 | 0.00020875 |
| UGDH-AS1 | 4.589601794 | 0.00021854 |
| LINC00402 | 4.601865883 | 0.00022914 |
| C3orf35 | 4.468426935 | 0.00023001 |
| LINC01579 | 4.256315735 | 0.0002403 |
| MYCBP2-AS1 | 4.548568838 | 0.00025733 |
| DELEC1 | 3.602549206 | 0.00028226 |
| LINC00278 | 6.894217597 | 0.00029115 |
| FBXL19-AS1 | 3.768403803 | 0.00030222 |
| LINC01311 | 4.112906482 | 0.00033686 |
| LINC00852 | 4.520716464 | 0.0003427 |
| CERNA1 | 4.605708804 | 0.00035313 |
| LINC01277 | 5.106508917 | 0.00036568 |
| ZNF346-IT1 | 4.577051028 | 0.00037238 |
| PEG13 | 4.301598895 | 0.0003823 |
| MIR3142HG | 2.224597988 | 0.00038649 |
| LINC00885 | 4.253016451 | 0.00038665 |
| LINC02573 | 5.014573259 | 0.00039122 |
| BCL2L1-AS1 | 4.669943177 | 0.00039147 |
| SERPINB9P1 | 1.915911892 | 0.00041254 |
| STARD4-AS1 | 5.214648525 | 0.00041822 |
| DNAH10OS | 4.258297884 | 0.00050728 |
| UBAC2-AS1 | 4.304759113 | 0.00051625 |
| LINC02345 | 4.383714711 | 0.00052857 |
| TMEM252-DT | 4.82912272 | 0.00054689 |
| PRR34 | 4.426555428 | 0.00056071 |
| CLRN1-AS1 | 4.668891565 | 0.00060317 |
| CCDC26 | 4.258597949 | 0.00060719 |
| ZBTB20-AS4 | 2.639625979 | 0.00063723 |
| ZFY-AS1 | 5.327582309 | 0.00064148 |
| CDC42-IT1 | 4.59501032 | 0.00064221 |
| LINC00665 | 3.89392184 | 0.00065759 |
| ZBED3-AS1 | -2.970946156 | 0.00070581 |
| TYMSOS | 4.346108084 | 0.00071892 |
| EXTL3-AS1 | 4.534608818 | 0.0007441 |
| GPRC5D-AS1 | 4.193091321 | 0.00076303 |
| LINC00664 | 5.195463705 | 0.00077518 |
| RAB11B-AS1 | 2.021302417 | 0.00078993 |
| LINC00682 | -5.851758316 | 0.00080759 |
| CDC42-AS1 | 3.668332048 | 0.00081557 |
| CYP4A22-AS1 | 4.620903548 | 0.00091978 |
| LINC02073 | 5.052718715 | 0.00096258 |
| GTSE1-DT | 4.31432024 | 0.0009725 |
| SSBP3-AS1 | 4.150199133 | 0.0009839 |
| LINC02185 | 5.230995029 | 0.00098505 |
| LINC01004 | 1.419779386 | 0.0009909 |
| ID2-AS1 | 4.240152404 | 0.00099667 |
| CPEB2-DT | 4.812326399 | 0.0010017 |
| LINC02693 | 3.150917009 | 0.00101897 |
| ST8SIA6-AS1 | 4.29270402 | 0.00108151 |
| LINC00893 | 4.727330253 | 0.0010918 |
| MORF4L2-AS1 | 4.108497444 | 0.00110743 |
| LINC00598 | 4.063586319 | 0.00118233 |
| BDNF-AS | 4.059381298 | 0.00118924 |
| ALMS1-IT1 | 4.791164653 | 0.00122807 |
| SRD5A3-AS1 | 3.652428053 | 0.00137853 |
| ADD3-AS1 | 4.36442829 | 0.00138722 |
| LINC01252 | 4.664026365 | 0.00140577 |
| PRKAR2B-AS1 | 4.560682851 | 0.00152093 |
| LIMS1-AS1 | 3.984820682 | 0.00152903 |
| POT1-AS1 | 4.082636102 | 0.00165489 |
| CASC15 | 2.973421546 | 0.00170647 |
| PSPC1-AS2 | 4.670424568 | 0.00178244 |
| LINC01736 | 4.033511791 | 0.0018898 |
| LINC02295 | 4.47356085 | 0.00189927 |
| DHRSX-IT1 | 4.222408546 | 0.0019943 |
| CAMTA1-DT | 4.34033402 | 0.00213012 |
| LRIG2-DT | 4.202833989 | 0.00217985 |
| FLNB-AS1 | -2.609956215 | 0.00218803 |
| LINC01837 | -5.88585327 | 0.00221695 |
| GNG12-AS1 | 4.498743458 | 0.00225802 |
| LINC02427 | 3.924886135 | 0.00233914 |
| PLAC4 | 4.54531147 | 0.00246207 |
| LINC02367 | 3.666805608 | 0.00247878 |
| BRWD1-IT1 | 4.150342169 | 0.00250342 |
| LINC02212 | 4.011444746 | 0.00257468 |
| TMEM202-AS1 | 4.291904973 | 0.00260941 |
| PARTICL | 3.942135215 | 0.00269236 |
| LINC02324 | 3.823245846 | 0.00274521 |
| LINC01376 | 4.344351366 | 0.00278781 |
| LINC02595 | 4.303429525 | 0.00289812 |
| CRTC3-AS1 | 4.326285349 | 0.00308675 |
| SEC24B-AS1 | 3.645536294 | 0.00318917 |
| DTX2P1-UPK3BP1-PMS2P11 | 3.893520609 | 0.003226 |
| REV3L-IT1 | 3.635276968 | 0.00363796 |
| LINC01569 | 4.335526057 | 0.00375382 |
| DLEU2L | 4.50960213 | 0.00388113 |
| ST3GAL6-AS1 | 2.83942427 | 0.00392425 |
| MSC-AS1 | 2.170326438 | 0.00399564 |
| ZRANB2-AS1 | 4.420755799 | 0.00399693 |
| MYLK-AS1 | 4.24250407 | 0.00408028 |
| LINC02453 | 4.242757287 | 0.00471093 |
| CNNM3-DT | 4.137915236 | 0.0048303 |
| LINC01465 | 3.490335003 | 0.00498547 |
| LINC00920 | 4.171939113 | 0.00508693 |
| LYST-AS1 | 4.185208219 | 0.00514746 |
| MMADHC-DT | 4.226258182 | 0.00524277 |
| HCG11 | -0.825192632 | 0.00525738 |
| LINC01852 | -4.440875605 | 0.00532513 |
| LINC01890 | 3.885080921 | 0.00532683 |
| LINC01545 | 3.729035262 | 0.00552627 |
| ITPR1-DT | 3.157430661 | 0.00595354 |
| RBM26-AS1 | 3.575574264 | 0.00608485 |
| PRKAR2A-AS1 | 3.736984056 | 0.00680089 |
| ZRANB2-AS2 | 4.557765282 | 0.00699329 |
| C2CD4D-AS1 | 4.304759331 | 0.00737817 |
| LINC01133 | 4.037078254 | 0.00738846 |
| LINC02664 | 4.002079541 | 0.00789833 |
| LINC01725 | 3.393364609 | 0.00814044 |
| PACERR | 4.473980944 | 0.00937955 |
| LINC02576 | 3.927308014 | 0.00962084 |
| LAMTOR5-AS1 | -1.201959922 | 0.00971843 |
| ZNF350-AS1 | 3.613586806 | 0.01003798 |
| MIR17HG | 1.625171927 | 0.01020513 |
| L3MBTL4-AS1 | 3.289859566 | 0.01180424 |
| MME-AS1 | 3.830674952 | 0.01189683 |
| SNCA-AS1 | 3.717624367 | 0.01259531 |
| PRSS30P | 4.150582908 | 0.01263476 |
| LINC01806 | 2.357020614 | 0.01347828 |
| PRNCR1 | 3.151984973 | 0.01380356 |
| MROCKI | 5.533118605 | 0.01389307 |
| TMEM92-AS1 | 3.58493215 | 0.01433104 |
| ZNF571-AS1 | 2.981838845 | 0.01446464 |
| MIR155HG | 1.175898468 | 0.01451969 |
| OSGEPL1-AS1 | 3.801530969 | 0.01478421 |
| LINC01088 | 3.623356098 | 0.01481082 |
| MIR9-3HG | 3.519932032 | 0.01535683 |
| TSC22D1-AS1 | 3.787578802 | 0.0171906 |
| H2AZ1-DT | 3.739658306 | 0.0172454 |
| GAS5-AS1 | 4.112167915 | 0.01772426 |
| LINC02770 | 4.237169136 | 0.01790171 |
| PKIA-AS1 | 4.006450328 | 0.01883678 |
| POLR2J4 | 0.843463276 | 0.01913698 |
| MIR194-2HG | 3.708647151 | 0.01920788 |
| LINC00607 | 2.018170763 | 0.01928609 |
| LINC02044 | 4.018920612 | 0.01954992 |
| LINC01344 | 4.002418263 | 0.02067941 |
| LINC01993 | 3.809039968 | 0.02117752 |
| LINC01891 | 3.805390719 | 0.02196008 |
| LINC01675 | 3.519865615 | 0.02249376 |
| LINC01825 | 3.666900614 | 0.02262629 |
| DUBR | 3.587309653 | 0.02459384 |
| PLCB1-IT1 | 3.916517915 | 0.02573947 |
| ITCH-AS1 | 3.572512708 | 0.02583493 |
| LINC00562 | 3.748651658 | 0.02652567 |
| DAAM2-AS1 | 3.949245416 | 0.02767596 |
| LINC01473 | 2.620361948 | 0.02921397 |
| LINC00865 | 3.572368442 | 0.02949062 |
| CABIN1 | -0.583753749 | 0.03143156 |
| TBL1XR1-AS1 | 3.530895881 | 0.03439693 |
| LINC02605 | 2.26968477 | 0.03475448 |
| TCL6 | 3.727326135 | 0.03533951 |
| LINC01474 | 3.967532881 | 0.03805523 |
| LINC00867 | -2.089803695 | 0.03894713 |
| CASC11 | 3.733933585 | 0.04050412 |
| LINC00504 | 3.582510025 | 0.04209941 |
| KIZ-AS1 | 3.10658765 | 0.04476544 |
| LINC01218 | 3.61207817 | 0.04524176 |
| CAPN15 | 0.791795901 | 0.04542511 |
| LINC01932 | 3.393659316 | 0.04554565 |
| ZNF341-AS1 | 3.390322472 | 0.04559593 |
