# Supplementary material for: lncRNA CARINH regulates expression and function of innate immune transcription factor IRF1 in macrophages
Source: Life Sci Alliance. 2025 Jan 7;8(3):e202403021. doi: 10.26508/lsa.202403021 (PMC11707381; doi:10.26508/lsa.202403021)
Supplement: Supplementary file 2 [file LSA-2024-03021_TableS2.docx]

**Supplemental Material**

Table S2. Rank-sum ordered lncRNA/mRNA pairs in MPV, IAV and CoV2

| **Rank** | **lncRNA** | **mRNA** |
| --- | --- | --- |
| 1 | GSEC | ST3GAL4 |
| 2 | LINC02422 | RESF1 |
| 3 | CARINH | IRF1 |
| 4 | CFAP58-DT | CFAP58 |
| 5 | LINC02471 | LRRK2 |
| 6 | LINC01506 | TMEM252 |
| 7 | ADAMTSL4-AS1 | ADAMTSL4 |
| 8 | RERE-AS1 | RERE |
| 9 | GSN-AS1 | GSN |
| 10 | CCR5AS | CCRL2 |
| 11 | RNF213-AS1 | RNF213 |
| 12 | BISPR | BST2 |
| 12 | BISPR | MVB12A |
| 13 | ITPK1-AS1 | ITPK1 |
| 14 | LINC01353 | CHIT1 |
| 15 | HIF1A-AS3 | HIF1A |
| 16 | BASP1-AS1 | BASP1 |
| 17 | KCNJ2-AS1 | KCNJ2 |
| 18 | PSMB8-AS1 | PSMB9 |
| 18 | PSMB8-AS1 | TAP1 |
| 18 | PSMB8-AS1 | PSMB8 |
| 19 | PATL2 | B2M |
| 19 | PATL2 | SPG11 |
| 20 | DLEU2 | TRIM13 |
| 20 | DLEU2 | KCNRG |
| 21 | EIF1B-AS1 | EIF1B |
| 22 | PSMD6-AS2 | PSMD6 |
| 23 | AOAH-IT1 | AOAH |
| 24 | HLA-F-AS1 | HLA-F |
| 25 | LINC00528 | BID |
| 26 | PCED1B-AS1 | PCED1B |
| 27 | PRKCQ-AS1 | PRKCQ |
| 28 | ILF3-DT | ILF3 |
| 29 | MKNK1-AS1 | MKNK1 |
| 30 | IL10RB-DT | IL10RB |
| 31 | A2M-AS1 | A2M |
| 32 | LINC00685 | GTPBP6 |
| 33 | TSPOAP1-AS1 | TSPOAP1 |
| 33 | TSPOAP1-AS1 | SUPT4H1 |
| 34 | SNHG1 | SLC3A2 |
| 35 | SNHG29 | TRPV2 |
| 36 | VIPR1-AS1 | VIPR1 |
| 37 | CEROX1 | LMF1 |
| 38 | HOXB-AS1 | HOXB2 |
