# Supplementary material for: lncRNA CARINH regulates expression and function of innate immune transcription factor IRF1 in macrophages
Source: Life Sci Alliance. 2025 Jan 7;8(3):e202403021. doi: 10.26508/lsa.202403021 (PMC11707381; doi:10.26508/lsa.202403021)
Supplement: Supplementary file 3 [file LSA-2024-03021_TableS3.docx]

**Supplemental Material**

**Table S3. Pearson correlation of mRNA and lncRNA (Log_10_-transformed CPM)**

| **mRNA vs. lncRNA** | **Rho** | **P-value** |
| --- | --- | --- |
| ST3GAL vs. GSEC | 0.8978 | <0.0001 |
| RESF vs.LINC02422 | 0.6524 | <0.0001 |
| IRF1 vs.CARINH | 0.9556 | <0.0001 |
| CFAP58 vs. CFAP58-DT | 0.7533 | <0.0001 |
| LRRK2 vs. LINC02471 | 0.6193 | <0.0001 |
| TMEM252 vs. LINC01506 | 0.7314 | <0.0001 |
| ADAMTSL4 vs.ADAMTSL4-AS1 | 0.8365 | <0.0001 |
| RERE vs. RERE-AS1 | 0.8918 | <0.0001 |
| GSN vs. GSN-AS1 | 0.4713 | 0.0019 |
| CCRL2 vs. CCR5AS | 0.8915 | <0.0001 |
| RNF213 vs. RNF213-AS1 | 0.5487 | 0.0002 |
| BST2 vs. BISPR | 0.7869 | <0.0001 |
| MVB12A vs.BISPR | 0.8282 | <0.0001 |
| ITPK1 vs. ITPK1-AS1 | 0.3859 | 0.0127 |
| CHIT1 vs. LINC01353 | -0.0216 | 0.8962 |
| HIF1A vs. LINC01353 | 0.5220 | 0.0009 |
| BASP1 vs. BASP1-AS1 | 0.8082 | <0.0001 |
| KCNJ2 vs. KCNJ2-AS1 | 0.7545 | <0.0001 |
| PSMB8 vs. PRKCQ-AS1 | -0.6839 | <0.0001 |
| PSMB9 vs. PRKCQ-AS1 | -0.6302 | <0.0001 |
| TAP1 vs. PSMB8-AS1 | 0.8556 | <0.0001 |
| B2M vs. PATL2 | 0.0829 | 0.606 |
| SPG11 vs. PATL2 | 0.4653 | 0.002 |
| KCNRG vs. DLEU2 | 0.8063 | <0.0001 |
| TRIM13 vs. DLEU2 | 0.8487 | <0.0001 |
| EIF1B vs. EIF1B-AS1 | 0.2552 | 0.111 |
| PSMD6 vs. PSMD6-AS2 | 0.2619 | 0.098 |
| AOAH vs. AOAH-IT1 | 0.6719 | <0.0001 |
| HLA-F vs. HLA-F-AS1 | 0.2434 | 0.1252 |
| BID vs. LINC00528 | 0.5196 | 0.0005 |
| PCED1B vs. PCED1B-AS1 | 0.8304 | <0.0001 |
| PRKCQ vs. PRKCQ-AS1 | 0.7159 | <0.0001 |
| ILF3 vs. ILF3-DT | 0.5503 | 0.0002 |
| MKNK1 vs. MKNK1-AS1 | 0.6156 | <0.0001 |
| IL10RB vs. IL10RB-DT | 0.8478 | <0.0001 |
| A2M vs. A2M-AS1 | 0.5524 | 0.0003 |
| GTPBP6 vs. LINC00685 | 0.5188 | 0.0005 |
| SUPT4H1 vs. SNHG29 | -0.2065 | 0.1952 |
| TSPOAP1 vs. TSPOAP1-AS1 | -0.0491 | 0.7604 |
| SLC3A2 vs. SNHG1 | -0.1060 | 0.5094 |
| TRPV2 vs. SNHG29 | -0.4216 | 0.0060 |
| VIPR1 vs. VIPR1-AS1 | 0.4687 | 0.0068 |
| LMF1 vs. CEROX1 | 0.2807 | 0.0755 |
| HOXB2 vs.HOXB-AS1 | 0.7257 | <0.0001 |
