# Supplementary material for: lncRNA CARINH regulates expression and function of innate immune transcription factor IRF1 in macrophages
Source: Life Sci Alliance. 2025 Jan 7;8(3):e202403021. doi: 10.26508/lsa.202403021 (PMC11707381; doi:10.26508/lsa.202403021)
Supplement: Supplementary file 4 [file LSA-2024-03021_TableS4.docx]

**Supplemental Material**

Table S4. Table of oligonucleotides used in this study.

|  |  | Primer | Sequence |
| --- | --- | --- | --- |
| qPCR | human | hGAPDH_F | GAAGGTGAAGGTCGGAGTC |
|  |  | hGAPDH_R | GAAGATGGTGATGGGATTTC |
|  |  | hACTB_F | TGCGTTACACCCTTTCTTGA |
|  |  | hACTB_R | AAAGCCATGCCAATCTCATC |
|  |  | hHPRT1_F | ACCCCACGAAGTGTTGGATA |
|  |  | hHPRT1_R | AAGCAGATGGCCACAGAACT |
|  |  | hCARINH_F | AGAAAATGGACTCCCTGGCTG |
|  |  | hCARINH_R | GCTACAAACTCTGGCACCCAT |
|  |  | hCARINH-V1_F | CAGCCAGACAGGTCTGAGAAA |
|  |  | hCARINH-V1_R | GCCTCAAGATGGGTGTCCAA |
|  |  | hCARINH-V2_F | GGTTGGAGAGAGAAACGCGA |
|  |  | hCARINH-V2_R | CACTCGGCCATCGCTAAGAT |
|  |  | hCARINH-V3_F | TCTGCCATATGCCTGGTCAC |
|  |  | hCARINH-V3_R | CATCCTGAGCCTTCGACTCC |
|  |  | hIRF1_F | ACATGCAGGACTTGGAGGTG |
|  |  | hIRF1_R | AACTTCCACTGGGATGTGCC |
|  |  |  |  |
| qPCR | mouse | mGAPDH_F | CATCACTGCCACCCAGAAGACTG |
|  |  | mGAPDH_R | ATGCCAGTGAGCTTCCCGTTCAG |
|  |  | mHPRT_F | CTGGTGAAAAGGACCTCTCGAAG |
|  |  | mHPRT_R | CCAGTTTCACTAATGACACAAACG |
|  |  | mCarinh_F | GAGGTGAGGGGAGAGGAGAG |
|  |  | mCarinh_R | GTCCAGCCTCCTGACATCAC |
|  |  | mIRF1_F | GTACCTACACCGCAACTCCG |
|  |  | mIRF1_R | TGATTGGCATGGTGGCTTTG |
|  |  |  |  |
| qPCR | influenza | PR8_F | CCACAACGGAAAACTATGTAG |
|  |  | PR8_R | CTGGAAGCAGTGGGTC |
|  |  |  |  |
| Genotyping | mouse | GT1_F | GCTGTGTTCGCTCATGGTGC |
|  |  | GT2_R | CAGGACTGCAATATCCTCAGCGTG |
|  |  |  |  |
| ChIRP | *CARINH* | POOL_1 | ttcctccaataggctacaaa |
|  |  | POOL_2 | atctgtacaacttccaggtg |
|  |  | POOL_1 | aaagcacacaaccttgacct |
|  |  | POOL_2 | tgggatgactctttctcttg |
|  |  | POOL_1 | tgatgaccacagcagctaca |
|  |  | POOL_2 | aagaaatgctaaggtgggcc |
|  |  | POOL_1 | ccccacagaaagaaagtcga |
|  |  | POOL_2 | gtatccaaggactctgggtc |
|  |  | POOL_1 | aaaaggagccagatcccaag |
|  |  | POOL_2 | tctggtagcataagaggtca |
|  |  | POOL_1 | gctagatgtgtttaagccaa |
|  |  | POOL_2 | tgatagcccaaggaggaaga |
|  |  | POOL_1 | cctttatccttgagagatgg |
|  |  | POOL_2 | aaaagcagctgtaagccagg |
|  |  | POOL_1 | agagtcgatcatctcagcaa |
|  |  | POOL_2 | acgttaacacagaggctctt |
|  |  | POOL_1 | gctgggacatcaacaaggat |
|  |  | POOL_2 | caaagtctgtggtctctgac |
|  |  | POOL_1 | tccagagtggtctgtttaag |
|  |  | POOL_2 | tgttcaccatccaagtctag |
|  |  | POOL_1 | cttgaacggtctgactcaga |
|  | LacZ | LacZ_1 | ctaatccgagccagtttacc |
|  |  | LacZ_1 | tttcgctcgggaagacgtac |
|  |  | LacZ_1 | atcagttgctgttgactgta |
|  |  | LacZ_1 | taattccgccgatactgacg |
|  |  | LacZ_1 | tcgatgcggccttaagaaaa |
|  |  | LacZ_1 | tctgtcgtttcctttctctg |
|  |  | LacZ_1 | aattttccggtttaaggcgt |
|  |  | LacZ_1 | cacgcacgttgtgatatgta |
|  |  | LacZ_1 | tcagtgtcgctgatttgtat |
